# Identification of CD24 as a Cancer Stem Cell Marker in Human Nasopharyngeal Carcinoma

**DOI:** 10.1371/journal.pone.0099412

**Published:** 2014-06-23

**Authors:** Chun-Hung Yang, Hui-Ling Wang, Yi-Sheng Lin, K. P. Shravan Kumar, Hung-Chi Lin, Chih-Jung Chang, Chia-Chen Lu, Tsung-Teng Huang, Jan Martel, David M. Ojcius, Yu-Sun Chang, John D. Young, Hsin-Chih Lai

**Affiliations:** 1 Graduate Institute of Biomedical Sciences, College of Medicine, Chang Gung University, Taoyuan, Taiwan, Republic of China; 2 Division of Applied Toxicology, Taiwan Agricultural Chemicals and Toxic Substances Research Institute, Council of Agriculture, Executive Yuan, Taichung, Taiwan, Republic of China; 3 Department of Medical Biotechnology and Laboratory Sciences, College of Medicine, Chang Gung University, Taoyuan, Taiwan, Republic of China; 4 Department of Laboratory Medicine, Linkou Chang Gung Memorial Hospital, Taoyuan, Taiwan, Republic of China; 5 Department of Microbiology and Immunology, College of Medicine, Chang Gung University, Taoyuan, Taiwan, Republic of China; 6 Department of Respiratory Therapy, Fu Jen Catholic University, Taipei, Taiwan, Republic of China; 7 Center for Molecular and Clinical Immunology, College of Medicine, Chang Gung University, Taoyuan, Taiwan, Republic of China; 8 Research Center of Bacterial Pathogenesis, College of Medicine, Chang Gung University, Taoyuan, Taiwan, Republic of China; 9 Department of Molecular Cell Biology and Health Sciences Research Institute, University of California Merced, Merced, California, United States of America; 10 Molecular Medicine Research Center, College of Medicine, Chang Gung University, Taoyuan, Taiwan, Republic of China; 11 Laboratory of Cellular Physiology and Immunology, Rockefeller University, New York, New York, United States of America; 12 Biochemical Engineering Research Center, Ming Chi University of Technology, Taipei, Taiwan, Republic of China; Okayama University, Japan

## Abstract

Cancer stem cells (CSCs) represent a unique sub-population of tumor cells with the ability to initiate tumor growth and sustain self-renewal. Although CSC biomarkers have been described for various tumors, only a few markers have been identified for nasopharyngeal carcinoma (NPC). In this study, we show that CD24^+^ cells isolated from human NPC cell lines express stem cell genes (*Sox2*, *Oct4*, *Nanog*, *Bmi-1*, and *Rex-1*), and show activation of the Wnt/β-catenin signaling pathway. CD24^+^ cells possess typical CSC characteristics that include enhanced cell proliferation, increased colony and sphere formation, maintenance of cell differentiation potential in prolonged culture, and enhanced resistance to chemotherapeutic drugs. Notably, CD24^+^ cells produce tumors following inoculation of as few as 500 cells in immunodeficient NOD/SCID mice. CD24^+^ cells further show increased invasion ability *in vitro*, which correlates with enhanced expression of matrix metalloproteinase 2 and 9. In summary, our results suggest that CD24 represents a novel CSC biomarker in NPC.

## Introduction

NPC incidence rates vary throughout the world but are highest in Southeast Asia, especially in the Chinese province of Guangdong as well as in Hong Kong and Taiwan [1]. Intermediate NPC incidence is observed in countries of the Mediterranean basin along with Greenland and Alaska, while a low incidence is found in most Western countries [2]. In Taiwan, the incidence for females and males in 2007 was 2.93 and 8.41 cases per 100,000 individuals, respectively [3]. NPC usually shows relatively high sensitivity to radiation, and radiotherapy thus remains the major treatment for early-stage NPC [4], [5]. Although the combination of radiotherapy and chemotherapy has improved treatment outcomes for patients with advanced-stage NPC, cancer recurrence still occurs frequently [6].

Identification and characterization of tumor-initiating cells, also called cancer stem cells (CSCs), has revealed cellular mechanisms that could account for the refractoriness to treatment and dormant behavior of many tumors [7]. The progenitor cell hypothesis of cancer development suggests that only a small sub-population of cells within a tumor (the CSCs) has stem-like properties and the ability to initiate new tumors [8]. CSCs may not only initiate primary tumors, but may also be responsible for recurrence following treatment, a phenomenon that may be due to the self-renewal capacity and inherent chemo- and radio-resistance of these cells [9]. CSCs have been identified and isolated from various tumors using specific cell surface markers, including CD44 for head and neck cancer [10], CD133 for prostate cancer [11], CD90 for liver tumor [12], CD24 for ovarian cancer [13], CD133 for brain tumor [14], CD44^+^CD24^−/low^ for breast cancer [15], and CD133 for lung cancer [16].

Although specific biomarkers have been used to isolate CSCs, each type of tumor shows specific cell surface markers [17]. To isolate potential sub-populations of CSCs from tumors in which CSC biomarkers have not yet been identified, researchers have made use of the ability of CSCs to exclude the dyes, Hoechst 33342 or PKH26 [18]–[20]. Using this method, a sub-population of cells displaying CSC characteristics has been identified in NPC [21], [22]. However, isolation of NPC CSCs using cell surface makers has not been possible so far, with the exception of CD44, which was identified in a previous report [23]. In the present study, we describe the isolation of a sub-population of CD24^+^ cells from two NPC cell lines, and show that the isolated cells express stem cell gene markers. The isolated CD24^+^ cells exhibit typical CSC characteristics, and initiate tumor growth in non-obese diabetic/severe combined immunodeficiency (NOD/SCID) mice at a low cell number. Higher invasion ability and increased expression of metalloproteinase 2 and 9 (MMP2 and MMP9) were also observed. Our observations suggest that CD24^+^ cells represent a CSC biomarker in human NPC. These findings may lead to the development of novel therapeutic strategies to target NPC CSCs in human patients.

## Results

### Isolation of a sub-population of CD24^+^ cells from NPC cell lines

**Figure 1:**
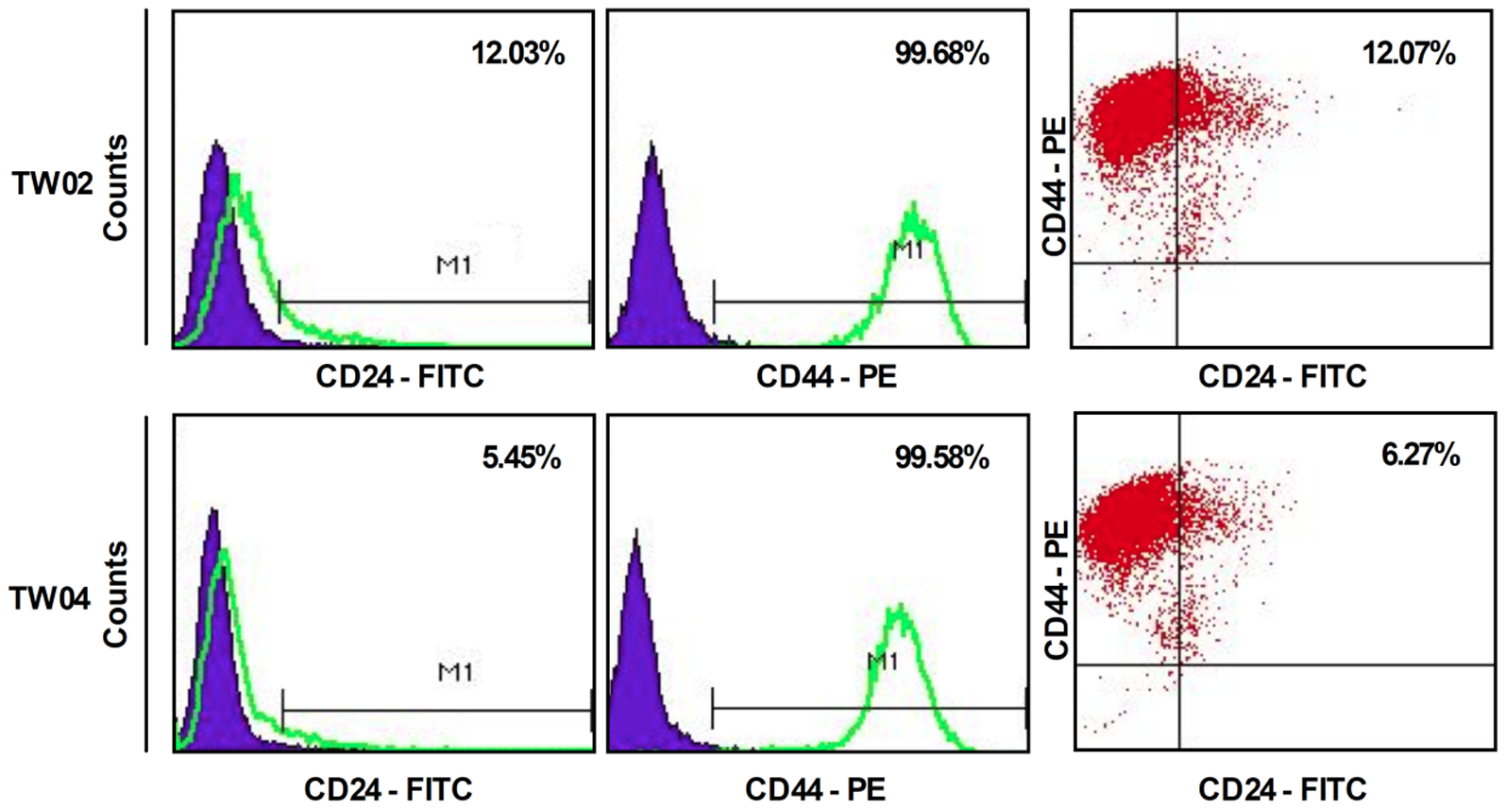
Flow cytometry analysis of CD24 and CD44 cell surface markers in NPC cell lines. Flow cytometry analysis of CD24^+^ and CD44^+^ sub-populations of TW02 and TW04 cells. 1×10^6^ cancer cells were collected and stained with anti-human CD24-fluorescein isothiocyanate (FITC) and/or anti-human CD44-phycoerythrin (PE) antibodies. Isotype-matched human antibodies served as control.

To characterize cells with specific cell surface markers, we analyzed several NPC cell lines using flow cytometry and monoclonal antibodies that recognize cell surface biomarkers. We determined the percentage of cells harboring one of 12 surface markers specific for stem cells [24], [25] or cancer cells [26] (Table 1). CD24 was found on 12.03% of cultured TW02 cells and on 5.45% of TW04 cells, while it was found on a lower percentage of cells for the other NPC cell lines tested (Table 1 and Figure 1). The percentage of cells harboring the other 12 markers was highly uneven, with values ranging from 0 to 99.99% (Table 1). Consistent with a previous study in which CD44 was identified as a CSC biomarker in NPC [23], our results showed that the vast majority of CD24^+^ cells were also CD44+ (Figure 1). We therefore focused our attention on CD24^+^ cells as potential CSC candidates.

**Table 1:** Percentage of cells expressing cancer cell and stem cell markers in human NPC cell lines.

	Marker	TW02	TW04	HK1	CG1	TW039	TW076
1	CD15	0	0	0	0	0	0
2	CD24	12.03	5.45	0.86	2.16	1.92	1.37
3	CD30	0	0	0	0	0.01	0
4	CD33	96.52	34.88	95.76	30.23	94.67	0.40
5	CD34	0	0	9.18	4.17	3.68	0
6	CD38	80.87	2.14	93.66	23.20	88.57	0
7	CD44	99.68	99.58	16.30	88.30	97.30	12.70
8	CD45	0	0.12	0	0.28	0.10	0.06
9	CD54	99.99	99.99	99.60	7.55	93.00	70.00
10	CD59	99.99	17.82	100.0	33.68	100.0	63.00
11	CD90	30.36	1.73	56.23	55.75	71.20	0.13
12	CD117	89.85	6.15	93.21	15.09	44.95	0.13

The values shown represent the percentage of cells expressing the indicated cell surface protein marker in the total cell population as assessed by flow cytometry.

### Expression of stem cell genes and activation of the Wnt/β-catenin pathway in CD24^+^ cells

**Figure 2:**
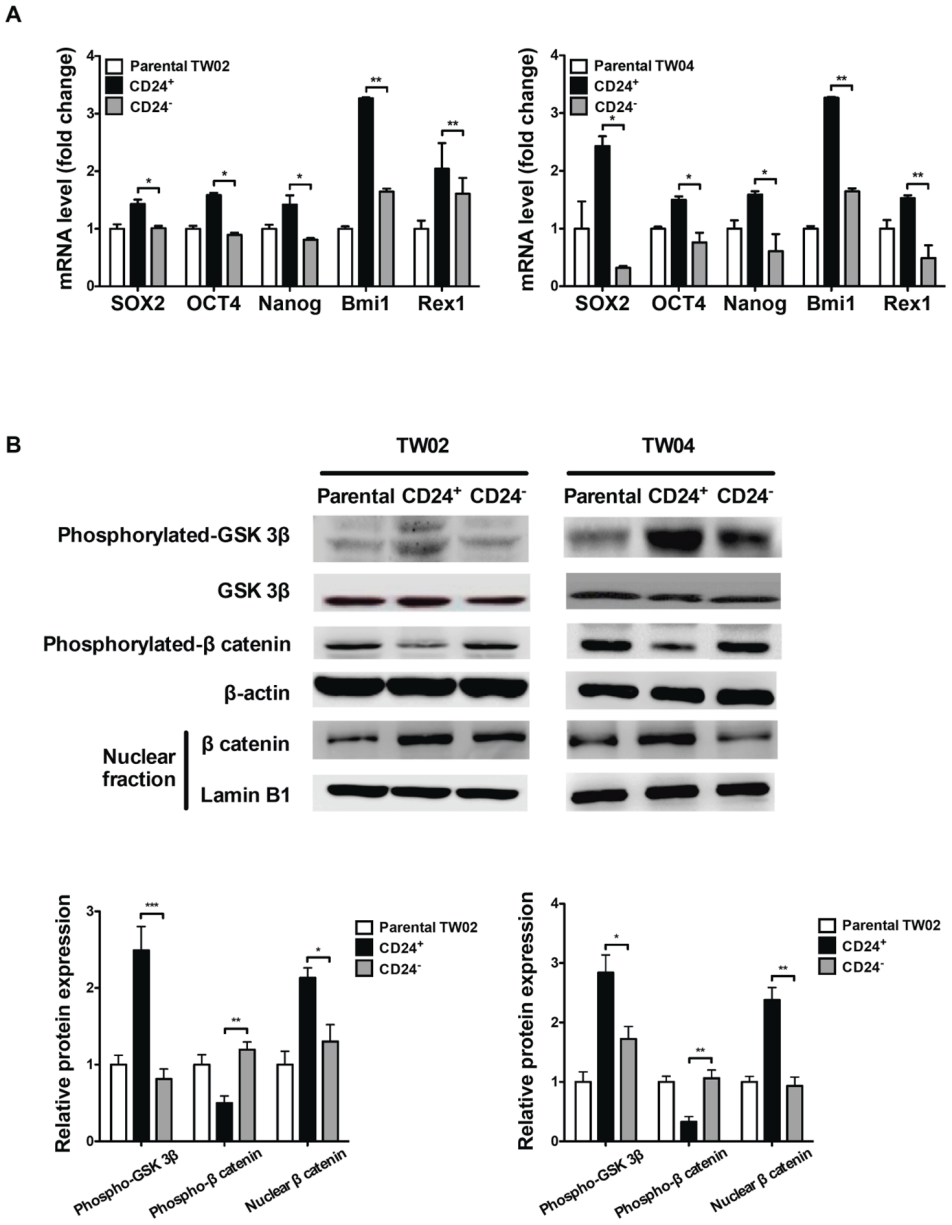
Expression levels of stem cell genes and activation of the Wnt/β-catenin pathway in CD24^+^ cells. (**A**) mRNA expression levels of *Sox2*, *Oct4*, *Nanog*, *Bmi-1* and *Rex-1* in parental, CD24^+^ and CD24^−^ cells from the NPC cell lines, TW02 (left) and TW04 (right), was evaluated using quantitative RT-PCR analysis. The results shown represented the average of three independent experiments. *: *p*<0.05, **: *p*<0.01. (**B**) Western blots analysis of phosphorylated-GSK, GSK, phosphorylated-β-catenin, β-catenin and β-actin in whole cell lysates, and of β-catenin and lamin B1 in nuclear fractions of parental, CD24^+^, and CD24^−^ cells isolated from the TW02 and TW04 cell lines. Quantitative result was calculated by ImageJ software. *: *p*<0.05, **: *p*<0.01, ***: *p*<0.001.

To examine whether CD24^+^ cells have intrinsic stem cell properties, we analyzed the expression of five embryonic stem cell genes (*Sox2* [27], *Oct4* [28]–[30], *Nanog* [31], *Bmi-1* [32], and *Rex-1* [33]) in TW02 and TW04 cell lines which were used as representative NPC cells. As indicated in Figure 2A, CD24^+^ cells consistently expressed higher levels of the five stem cell genes than parental or CD24^−^ cells.

Previous reports have indicated that activation of the Wnt/β-catenin signaling pathway is crucial for the maintenance of CSCs in leukemia [34], breast cancer [35], melanoma [36], colorectal adenoma [37], and liver cancer [38]. This cellular pathway also regulates self-renewal, proliferation, and differentiation of cancer stem-like cells in gastric cancer [39]. Furthermore, in the Wnt pathway, β-catenin plays a crucial role in regulating invasion and metastasis of numerous tumors [40]. We thus examined the status of the Wnt/β-catenin pathway in the isolated CD24^+^ cells. Levels of phosphorylated β-catenin were significantly reduced in the cytosol of CD24^+^ cells, compared with parental or CD24^−^ cells (Figure 2B). In addition, levels of phosphorylated glycogen synthase kinase 3β (p-GSK-3β), which acts as a negative regulator of β-catenin, were higher in CD24^+^ cells. Concomitantly, increased levels of β-catenin were observed in nuclear fractions of CD24^+^ cells (Figure 2B), suggesting that the Wnt/β-catenin signaling pathway is activated in these cells.

### Enhanced proliferation and clone/sphere formation in CD24^+^ cells

**Figure 3:**
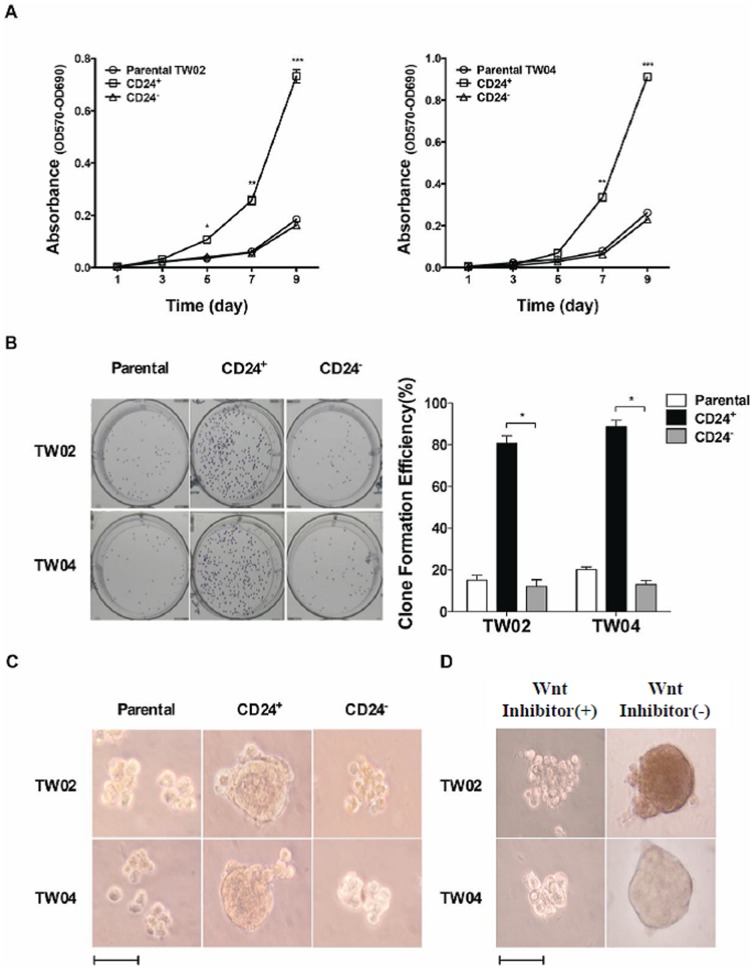
CD24^+^ cells show increased proliferation rate and enhanced clone and sphere formation. (**A**) Cell proliferation curves of parental, CD24^+^ and CD24^−^ cells from the TW02 (left) and TW04 (right) NPC cell lines cultured in complete DMEM for 9 days. The results shown represent the average of three independent experiments. *: *p*<0.05, **: *p*<0.01, ***: *p*<0.001. (**B**) Clone formation efficiency of parental, CD24^+^, and CD24^−^ cells, and the quantification analysis. *: *p*<0.05. (C) Sphere formation ability of parental, CD24^+^ and CD24^−^ cells cultured in DMEM supplemented with 20 ng/ml bFGF and 20 ng/ml EGF for 30 days. (D) Effect of the Wnt inhibitor on sphere formation by CD24^+^ cells in TW02 and TW04 cells. CD24^+^ cells were treated or not with the Wnt inhibitor ICG-001 (10 µM) for seven days prior to sphere formation analysis. Scale bars: 100 µm.

We measured the proliferation rate of CD24^+^ cells cultured in complete DMEM. Compared with parental and CD24^−^ cells, CD24^+^ cells showed enhanced proliferation, starting on the 5^th^ day of culture for TW02 cells and the 7^th^ day for TW04 cells, with doubling times of about 42 h, 42 h, and 30 h for parental TW02, CD24−, and CD24+ cells, respectively, and of 40 h, 40 h and 28 h for parental TW04, CD24−, and CD24+ cells (Figure 3A). The clone formation efficiency (CFE) of CD24^+^ cells was also determined. After 10 days of culture, when most clones were likely to contain over 50 cells, the calculated CFE values of TW02 cells were 15.1%, 80.8% and 12.2% for parental, CD24^+^, and CD24^−^ cells, respectively. For TW04 cells, CFE values of 20.2%, 90.3% and 13.0% were obtained for parental, CD24^+^, and CD24^−^ cells, respectively (Figure 3B). The formation of spheroid cell aggregates (or simply spheres), which is indicative of self-renewal ability, was further analyzed by culturing the cells in non-adherent conditions consisting of serum-free DMEM containing only epithelial growth factor (EGF) and basic fibroblast growth factor (bFGF). After 30 days of culture, CD24^+^ cells showed the highest number of spheres, reaching diameters between 100 and 200 µm, whereas parental cells and CD24^−^ cells formed significantly fewer spheres, with diameters below 100 µm (Figure 3C). These results indicate that CD24^+^ cells show enhanced proliferation and ability to form clones/spheres.

We also examined the effects of the Wnt inhibitor ICG-001 on the sphere formation ability of CD24^+^ cells. After treatment with the Wnt inhibitor for 7 days (10 µM), sphere size was considerably reduced by an average of 20% and 45% in TW02 and TW04 cells, respectively (Figure 3D), suggesting that the Wnt pathway may contribute to the CSC phenotype of CD24^+^ cells.

### Long-term differentiation potential of CD24^+^ cells

**Figure 4:**
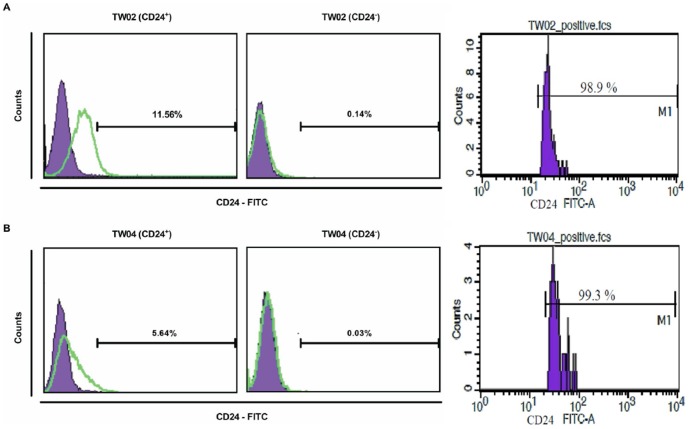
Cell differentiation potential of CD24^+^ cells. (**A**) Ten-day old cell masses originally cultured from CD24^+^ and CD24^−^ cell cultures in DMEM medium were harvested, and stained with anti-human CD24-fluorescein isothiocyanate (FITC) antibody followed by flow cytometry analysis. The percentages shown represent cells with CD24^+^ surface markers from either (**A**) TW02 or (**B**) TW04 cell lines. Flow cytometry analysis of TW02 and TW04 cells immediately after sub-fractionation (right panels).

A long-term culture of CSCs may increase its cell mass but also undergo asymmetric division to generate a low tumorigenic cell population with heterogeneous phenotypes [9]. To monitor the cell differentiation potential of CD24^+^ cells, we analyzed 10-day old cultures of CD24^+^ and CD24^−^ cells by flow cytometry. After culturing TW02 cells for 10 days, 11.56% of CD24^+^ cells remained CD24^+^, whereas 0.14% of the cells that were initially CD24^−^ expressed CD24 after culture (Figure 4A). Similarly, following culture of TW04 cells, 5.64% of CD24^+^ cells remained CD24^+^, while 0.03% of cells that were CD24^−^ initially were CD24^+^ after culture (Figure 4B). These results suggest that CD24^+^ cells show cell differentiation potential after prolonged culture, although the differentiation process may be only partial under these conditions.

### CD24^+^ cells show enhanced resistance to chemotherapeutic drugs

**Figure 5:**
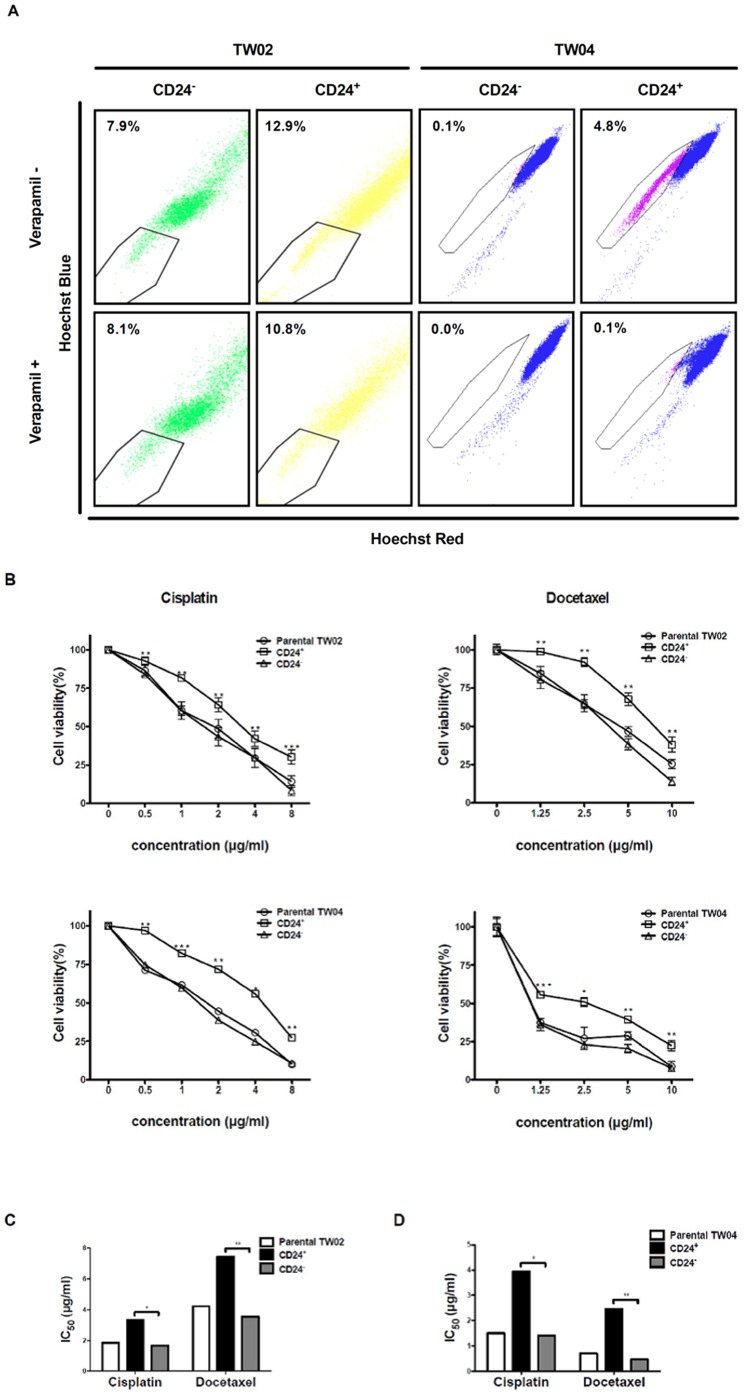
CD24^+^ cells show enhanced chemoresistance. (**A**) Sorted CD24^+^ and CD24^−^ cells from the TW02 and TW04 NPC cell lines were treated or not with 50 µM verapamil for 30 min prior to processing for the Hoechst 33342 dye exclusion assay as described in Materials and Methods. (**B**) Parental, CD24^+^, and CD24^−^ cells from the TW02 or TW04 cell lines were treated with various concentrations of cisplatin and docetaxel for 24 h, and cell viability was determined using the MTT assay. CD24^+^ cells showed higher viability than parental and CD24^−^ cells after treatment with either cisplatin or docetaxel. The results shown represented the average of three independent experiments. *: *p*<0.05, **: *p*<0.01, ***: *p*<0.001. IC_50_ values of cisplatin or docetaxel treatment against parental, CD24^+^, and CD24^−^ cells were shown for (**C**) TW02 and (**D**) TW04 cells lines.

Higher resistance to chemotherapeutic drugs represents a critical characteristic of CSCs [9]. Previous studies have reported that CSCs can exclude the DNA-binding dye, Hoechst 33342, due to enhanced expression of the ABC transporter, ABCG2 [41], [42]. To determine whether CD24^+^ cells exclude Hoechst 33342 better than parental or CD24^−^ cells, we performed the dye exclusion assay after treating TW02 cells with the ABC transporter inhibitor, verapamil [43]. As shown in Figure 5A, a larger population of Hoechst 33342-negative cells was observed for CD24^+^ cells (12.9%) compared with CD24^−^ cells (7.99%). Notably, verapamil treatment reversed the dye exclusion phenotype observed in CD24^+^ cells, while this treatment had no significant effect on CD24^−^ cells (Figure 5A). Similar results were obtained for the TW04 cell line (verapamil produced no significant effect in this case since almost no TW04 CD24^−^ cells were Hoechst 33342-negative). These observations suggest that CD24^+^ cells possess an enhanced ability to exclude dye.

To examine whether CD24^+^ cells show higher resistance to chemotherapeutic drugs, we treated the cells with either cisplatin or docetaxel, and assessed cell viability using the MTT assay. Cisplatin and docetaxel produced higher cytotoxic effects against parental or CD24^−^ cells compared with CD24^+^ cells (Figure 5B, *p*<0.01). For the TW02 cell line, the half maximal inhibitory concentration (IC_50_) of cisplatin against parental, CD24^+^, and CD24^−^ cells was 1.84, 3.32, and 1.66 µg/ml, respectively, while the IC_50_ values of docetaxel against parental, CD24^+^, and CD24^−^ cells was 4.23, 7.45, and 3.53 µg/ml, respectively (Figure 5C). For the TW04 cell line, cisplatin produced IC_50_ values of 0.70, 2.45, and 0.46 µg/ml against parental, CD24^+^, and CD24^−^ cells, respectively, whereas the IC_50_ of docetaxel was 1.50, 3.93, and 1.41 µg/ml against parental, CD24^+^, and CD24^−^ cells, respectively (Figure 5D).

**Figure 6:**
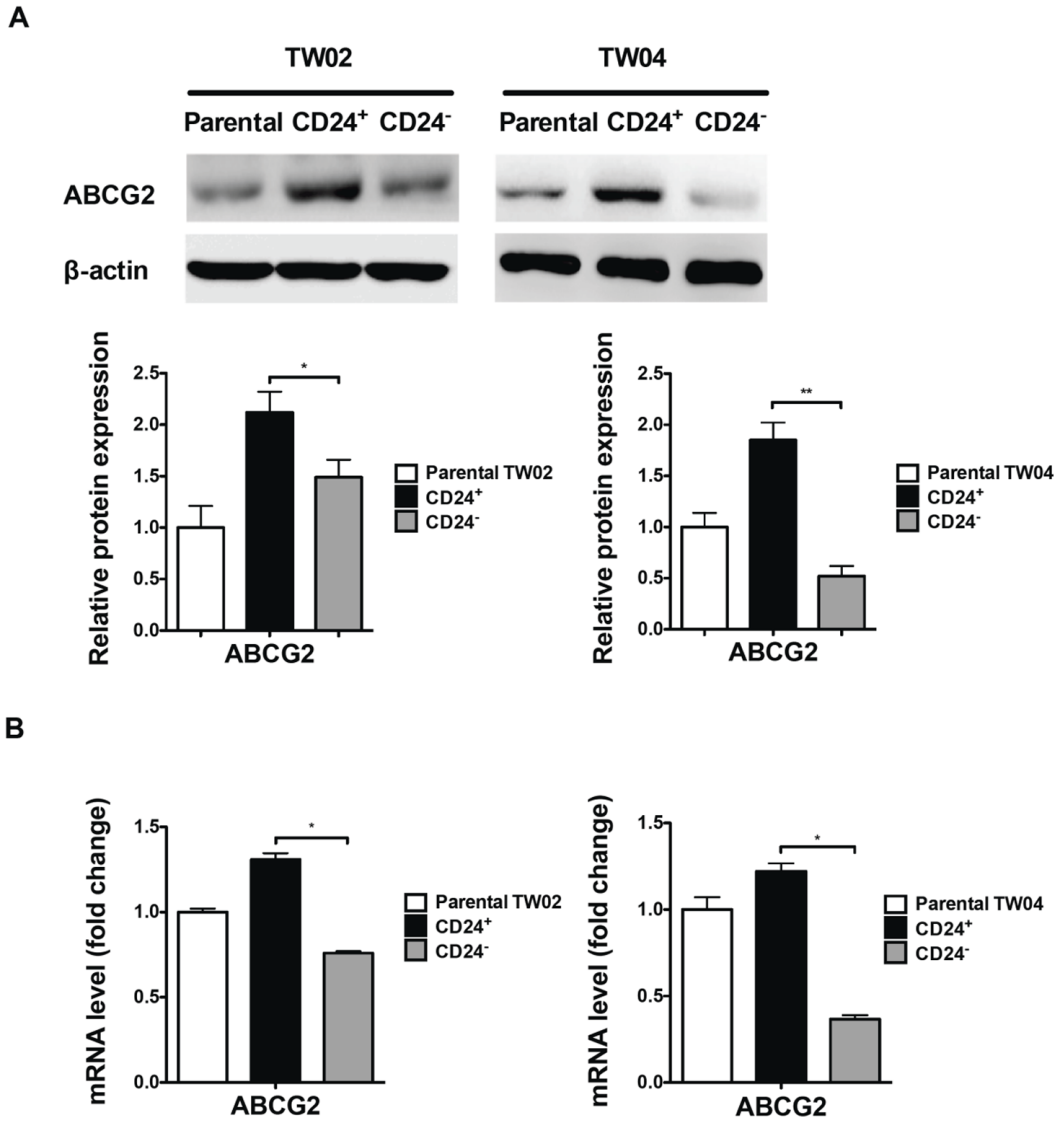
Cellular ABCG2 expression levels in parental, CD24^+^, and CD24^−^ cells. (**A**) Cellular ABCG2 protein levels in parental, CD24^+^, and CD24^−^ TW02 and TW04 cells were determined by Western blot analysis. Quantitative result was calculated by ImageJ software. *: *p*<0.05, **: *p*<0.01. (**B**) The mRNA expression level of ABCG2 in parental, CD24^+^, and CD24^−^ TW02 and TW04 cells was determined by quantitative RT-PCR. The results shown represent the average of three independent experiments. *: *p*<0.05, **: *p*<0.01.

We also observed that the protein levels of the ABC transporter ABCG2 were 1.42 and 2.12 fold higher in CD24^+^ cells than in parental and CD24^−^ cells, respectively (Figure 6A). Similarly, in TW04 cells, the protein levels of ABCG2 in CD24^+^ cells were 1.85 and 3.56 fold higher than in parental and CD24^−^ cells, respectively (Figure 6A). Consistent with these results, the mRNA expression level of *ABCG2* was higher in CD24^+^ cells than in parental or CD24^−^ cells (Figure 6B). These results indicate that CD24^+^ cells show enhanced chemoresistance, and that this phenotype may be attributed at least in part to enhance expression of ABCG2 transporter.

### A small number of CD24^+^ cells is sufficient to produce tumors in NOD/SCID mice

**Figure 7:**
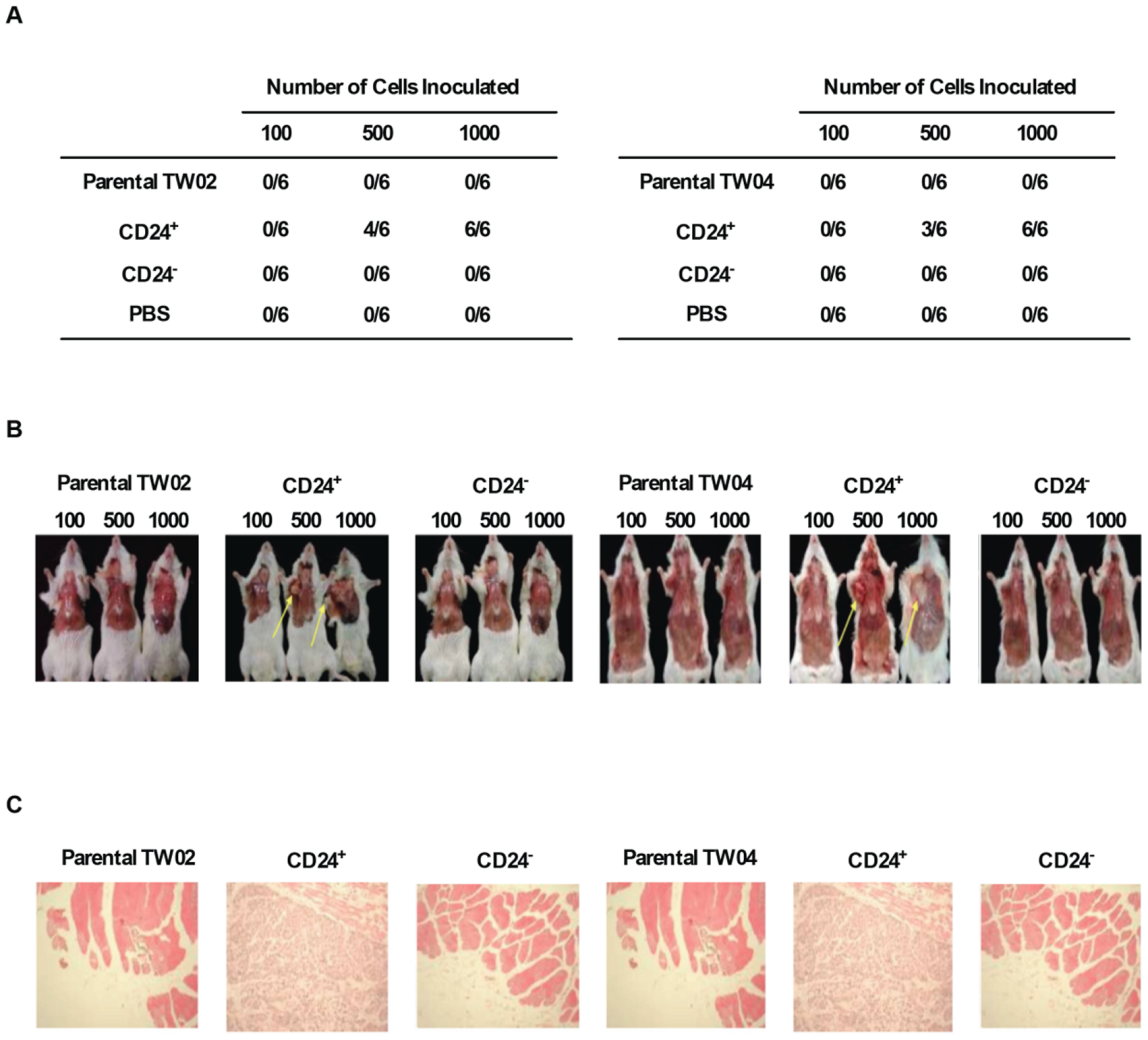
A low number of CD24^+^ NPC cells initiates tumor formation in NOD/SCID mice. (**A**) Formation of tumors following injection of CD24^+^ cells. Groups of six NOD/SCID mice were injected with 100, 500, or 1,000 freshly-sorted CD24^+^ or CD24^−^ cells from the TW02 (left) or TW04 (right) cell line. Mice injected with PBS were used as a negative control. Tumor formation was assessed 12 weeks after cell inoculation. (**B**) Mice injected with TW02 (left) or TW04 (right) cells were sacrificed for evaluation of tumor formation twelve weeks after inoculation. The arrows indicate the presence of tumors in mice injected with 500 or 1,000 CD24^+^ cells. (**C**) Tissue H&E staining results of TW02 (left) and TW04 (right) mice inoculated with 500 cells. Inoculation of as few as 500 CD24^+^ cells produced histological signs of tumors at the site of injection.

We tested the tumorigenic potential of CD24^+^ cells following injection in the axillary fossa of immunodeficient NOD/SCID mice. Twelve weeks after injection, tumors were detected in mice inoculated with either 500 or 1,000 CD24^+^ cells, but not in mice treated with 100 CD24^+^ cells (Figure 7A). In contrast, no tumors were detected in mice inoculated with ≤1,000 cells in the parental or CD24^−^ group (Figure 7A and 7B). Similarly, no tumors were detected in mice that received PBS as a negative control (Figure 7A). Further histological analysis of tissue sections stained with hematoxylin and eosin (H&E) confirmed the presence of nasopharyngeal squamous cell carcinoma and well-differentiated keratinizing squamous carcinoma at the site where CD24^+^ cells were initially injected (Figure 7C). Injection of a small number of CD24^+^ cells is thus sufficient to induce tumor formation in immunodeficient mice.

### Enhanced invasion potential and metalloproteinase production in CD24^+^ cells

**Figure 8:**
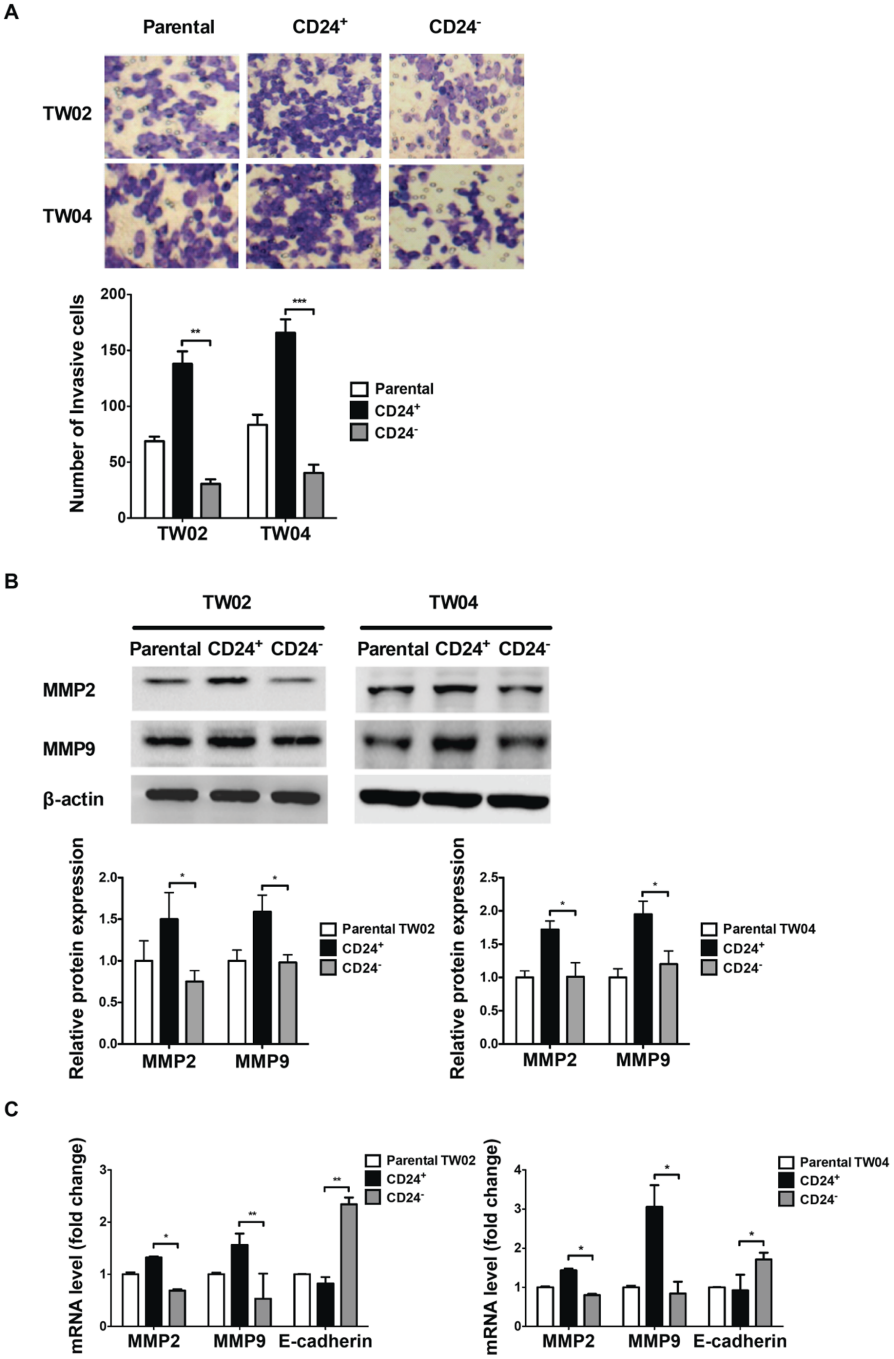
CD24^+^ cells show higher invasion ability and enhanced expression of MMP2 and MMP9. (**A**) The cell invasion assay was performed using the transwell chamber assay, as described in Materials and Methods. Matrigel membranes containing invading cells were observed by optical microscopy, and the cells were counted. The number of invading cells from each cell population was quantified. Results shown were obtained from three independent experiments. **: *p*<0.01, ***: *p*<0.001 (**B**) The levels of MMP2 and MMP9 protein produced by parental, CD24^+^, or CD24^−^ cells from the TW02 and TW04 cell lines were quantified by Western blotting analysis. Quantitative result was calculated by ImageJ software. *: *p*<0.05. (**C**) The mRNA expression levels of MMP2 and MMP9 in parental, CD24^+^, and CD24^−^ cells were determined by quantitative RT-PCR from the TW02 and TW04 cell lines. Results shown represent an average from three independent experiments. *: *p*<0.05, **: *p*<0.01.

In previous studies, many CSC populations showed increased invasion ability compared with non-CSCs [44], [45]. Using an in vitro invasion assay, we observed that CD24^+^ cells were more invasive than parental or CD24^−^ cells (Figure 8A). Given that metalloproteinases play an important role in cell invasion [46], we examined the level of MMP2 and MMP9 proteins in these cells. As shown in Figure 8B, CD24^+^ cell populations showed increased MMP2 and MMP9 protein levels compared with parental or CD24^−^ cells. Consistent with these results, we observed that CD24^+^ cells isolated from the TW02 or TW04 cell lines expressed higher levels of MMP2 and MMP9 mRNA than parental or CD24^−^ cells (Figure 8C). CD24^+^ cells also expressed lower E-cadherin mRNA levels than parental or CD24^−^ cells (Figure 8C). CD24^+^ cells thus show enhanced invasion and increased expression of MMP2 and MMP9 but reduced expression of E-cadherin.

## Discussion

The results of the present study suggest that CD24 represents a new CSC surface marker in NPC. The characteristics of CD24^+^ cell sub-populations isolated from the TW02 and TW04 NPC cell lines are similar to those reported previously for CSCs derived from NPC [21]–[23]. These characteristics include increased expression of genes involved in development and maintenance of stem cells, increased self-renewal and maintenance of cell differentiation capacity after prolonged culture, increased sphere formation ability (also observed in CD24^+^ cells isolated from the NPC cell lines HK1 and TW076; see Figure S1), increased Hoechst 33342 dye exclusion and chemoresistance, increased ability to initiate tumors in immunocompromised mice, and enhanced cell invasion.

The cell surface protein, CD24, is highly expressed in many human cancers [13], [48], [49]. CD24 expressed on cancer cells interacts with P-selectin found on endothelial cells, indicating that CD24 may bind to P-selectin and initiate rolling of cancer cells on the endothelium, which may be followed by initiation of metastasis [47]. In addition to NPC cells, CD24 is associated with CSCs of other tumors, such as ovarian [13] and pancreatic cancer [48]. For example, CD24^+^CD44^+^ESA^+^ pancreatic cancer cells possess CSC properties [48]. Recently, a similar finding was reported for ovarian CSCs; that is, a xenograft injection of 5,000 CD24^+^ cells produced tumors in nude mice, while injection of an equal number of CD24^−^ cells failed to do so [49]. In addition, CD24^+^ cancer cell colonies isolated from ovarian tumors of a human patient showed heterogeneity in proliferation rate, cell cycle distribution, and expression profile of genes and proteins, and demonstrated stem cell properties [49]. Furthermore, CD24^+^ stem-like cells detected in ovarian cancer also exhibited enhanced chemoresistance [50]. Notably, in breast cancer, the absence of CD24 combined with the presence of CD44 and EpCAM (CD24^−^CD44^+^EpCAM^+^) appears to be critical for the identification of breast CSCs [15]. A previous study has reported that CD44 represents a CSC biomarker in NPC [23]. Consistent with this finding, the CD24^+^ cells that we isolated from TW02 and TW04 NPC cell lines were mostly CD44^+^ (Figure 1B). Besides, the vast majority of CD24^+^ cells in the HK1 and TW076 cell lines were also CD44^+^ (Figure S2). Both CD24 and CD44 may thus be involved in the formation of CSCs in NPC, and the function of these proteins in the development and maintenance of CSCs warrants further investigation.

We observed that CD24^+^ cells isolated from the TW02 and TW04 NPC cell lines express higher mRNA levels of *Sox2*, *Oct-4*, *Nanog*, *Bmi-1*, and *Rex-1*, compared with parental or CD24^−^ cells. A similar phenomenon was also observed in HK1 and TW076 cell lines (Figure S3). These characteristics are similar to those reported for embryonic stem cells [27]–[33] and ovarian cancer stem-like cells [51]. This pattern of embryonic stem cell gene expression in CD24^+^ NPC CSCs indicates the presence of a conserved pattern of stem cell gene expression in embryonic stem cells and CSCs. In embryonic stem cells, co-expression of *Sox2*, *Oct-4*, *Nanog*, and *Rex-1* is essential for maintenance of pluripotency and self-renewal, and prevents cell differentiation along the trophoblast cell lineage [30], [33]. *Sox2* and *Oct-4* encode transcription factors that maintain self-renewal and pluripotency in the undifferentiated embryonic stem cell state by modulating genes that maintain permissive chromatin structure and DNA repair, and prevent apoptosis [52]. On the other hand, the expression of *Nanog*, which is also a transcription factor critically involved in self-renewal, is positively regulated by *Sox2* and *Oct-4* [53]. These transcription factors form a functional transcriptional regulation network that is critical for maintenance of pluripotency in embryonic stem cells [53]. Furthermore, the Polycomb group (PcG) gene *Bmi-1* silences *Hox* genes via modulation of chromatin structure during embryonic development in fruit flies, and may possibly enhance the proliferative activity of both normal and leukemic stem cells [32]. As the expression of *Bmi-1* is negatively regulated by *Pten*, which acts as a tumor suppressor gene [54], increased expression of *Bmi-1* may correlate with repression of *Pten* expression and induction of epithelial-mesenchymal transition, as well as increased motility and invasiveness of human nasopharyngeal epithelial cells [54]. Our results show that the CD24^+^ sub-population of NPC cells also expresses stem cell genes and exhibits characteristics of stem cell self-renewal and enhanced proliferation capacity.

The Wnt/β-catenin signaling pathway, which has been reported to regulate stem cell function and niche-stem cell interactions [56], enhances the expression of *Sox2* and *Oct-4* [55]. Moreover, reduced E-cadherin mRNA expression was observed by Gao et al. in CD24^+^ ovarian cancer stem cells [49]. These observations indicate that the loss of E-cadherin expression may allow β-catenin to re-localize to the nucleus and activate transcriptional activity [57]. In this study, increased phosphorylation of GSK-3β, reduced phosphorylation of β-catenin, and nuclear translocation of β-catenin were observed in CD24^+^ cells, compared with parental or CD24^−^ cells (Figure 2). These observations indicate that the Wnt/β-catenin signaling pathway is activated in CD24^+^ cells.

Our findings that CD24^+^ NPC cells are more invasive than parental or CD24^−^ NPC cells are consistent with results from previous studies on CSCs in pancreatic [58], lung [19], and prostate cancer [59]. Together with the increased invasion ability, cellular markers that characterize cell invasion, such as MMP2 and MMP9, were also increased at higher levels in CD24^+^ cells. MMP2 and MMP9 are involved in the degradation of the extracellular matrix, and an increase in expression of both enzymes induces cell invasion ability [60]. Zhou et al. suggested that genetic polymorphism in the MMP2 promoter may explain the increased susceptibility of Chinese individuals for developing NPC [61]. Microarray analysis reveals that MMP1 and MMP2 are the most significantly upregulated genes in NPC biopsies, compared with lymphohyperplasia, adenoid tissues, and head and neck cancer [62]. Furthermore, studies performed on NPC tumor biopsies or cell lines with enhanced invasiveness or epithelial-mesenchymal transitions also revealed enhanced expression of *Sox2*, *Oct-4*, and *Nanog* [63]–[66], similar to the findings reported here on CD24^+^ cells. These results indicate that CD24^+^ NPC CSCs may promote tumorigenesis and also initiate invasion and metastasis, and suggest that further experiments to confirm the presence of CD24^+^ CSCs in human NPC biopsies are warranted. In addition, whether the expression of stem cell and invasion genes is under the control of a common signaling pathway, such as the Wnt/β-catenin pathway, remains to be investigated.

In summary, our results show that CD24^+^ cells exhibit CSC characteristics in NPC cell lines. Strategies aimed at the eradication of CD24^+^ NPC cells may provide new and more effective treatment strategies against NPC.

## Materials and Methods

### Cell culture

NPC cell lines (the keratinizing squamous TW02 cell line; the undifferentiated TW04 cell line; differentiated squamous HK1 cells; poorly-differentiated squamous CG1 cells; and the keratinizing squamous TW039 and TW076 cell lines) were provided by Dr. Yu-Sun Chang (Chang Gung University) and maintained in Dulbecco's modified Eagle's medium (DMEM; Invitrogen, Grand Island, NY) supplemented with 10% fetal bovine serum (FBS), 100 units/ml penicillin, 100 µg/ml streptomycin and 25 µg/ml amphotericin B (Invitrogen). After sorting as described below, the cells were cultured in DMEM supplemented with 20 ng/ml bFGF (Sigma-Aldrich, St. Louis, MO), 20 ng/ml EGF (Sigma-Aldrich), 200 units/ml penicillin, 200 µg/ml streptomycin, and 50 µg/ml amphotericin B (Invitrogen). All cells were maintained in a humidified 5% CO_2_ incubator at 37°C.

### Flow cytometry analysis and sorting of NPC cells

The cells were analyzed by fluorescence-activated cell sorting (FACS) after reaching the logarithmic proliferation phase. Cells were digested with 0.25% trypsin-0.02% EDTA (Invitrogen), washed twice with calcium/magnesium-free PBS, and resuspended at a concentration of 1×10^6^ cells/ml in ice-cold PBS supplemented with 2% FBS. FITC-conjugated anti-human CD15, CD24, CD30, CD33, CD34, CD38, CD44, CD45, CD54, CD59, CD90, CD117 or CD133 antibody (BD Pharmingen, Becton Dickinson, Mountain View, CA) was then added at a final concentration of 1 µg/ml, and incubated for 30 min at 4°C in the dark with mixing. The cells were then washed twice with ice-cold PBS supplemented with 2% FBS, and were analyzed using the FACS Aria flow cytometer (Becton, Dickinson & Company, Mountain View, CA). Isotype-matched human antibodies (Becton, Dickinson & Company) were used as controls. Cells stained with FITC-conjugated anti-human CD24 were also sorted using the same flow cytometer.

### Tumor sphere formation assay

Six-well culture dishes were coated with 1.2% soft agar [22]. Parental and sorted cells were than counted and inoculated in triplicate at a density of 500 cells/well in DMEM supplemented with 20 ng/ml bFGF and 20 ng/ml EGF. Cells were observed for sphere formation every day. When the spheres grew and reached diameters of about 100 to 200 µm, images were taken using a Leica DM IL Inverted Contrasting Microscope (Leica Microsystems, Wetzlar, Germany) with a Nikon Coolpix P5100 digital camera (Nikon, Tokyo, Japan). In some experiments, the cells were treated with the Wnt inhibitor ICG-001 (R&D Systems, Minneapolis, MN; product #5439-DK-010) for seven days at 10 µM prior to analysis using the sphere formation assay.

### Clone formation assay

Parental and sorted cells were plated in triplicate at 300 cells/well in six-well plates, and cultured in DMEM supplemented with 10% FBS. After most cell colonies had expanded to >50 cells (about 10 days), cells were washed twice with PBS, fixed in ice-cold methanol for 30 min, and stained with 0.01% (w/v) crystal violet for 30 min at room temperature [22]. After a washing step, colony numbers containing >50 cells were counted and compared, and images were taken. The CFE values shown correspond to the ratio of clones obtained divided by the number of cells initially plated.

### Long-term cell differentiation assay

The cell differentiation assay was done 18 days after cell sorting. Sorted cells were cultured in DMEM supplemented with 20 ng/ml bFGF and 20 ng/ml EGF. When the desired cell numbers were obtained, cells were stained with FITC-conjugated anti-human CD24, followed by analysis by FACS to quantify the proportion of CD24^+^ cells in the sorted populations. Isotype-matched human antibodies served as control.

### Cell proliferation analysis

Sorted cells were incubated at 1,000 cells/well in 96-well plates, and cultured in triplicate in DMEM supplemented with 10% FBS to observe cell proliferation. Cell viability was assessed using the MTT Cell Growth Determination Kit (Sigma-Aldrich) according to the instructions provided by the manufacturer. Cell proliferation curves were drawn according to the results of background (OD_690_) and substrate (OD_570_) absorbance.

### Drug sensitivity assay

Parental and sorted cells were cultured in triplicate at 5,000 cells/well in 96-well plates, and cisplatin or docetaxel was added for 24 hours. Cell viability was determined as described above.

### Cell sub-population analysis

Sorted cells were cultured in DMEM supplemented with 20 ng/ml bFGF and 20 ng/ml EGF until they reached the desired cell number. Cells were then digested with 0.25% trypsin-0.02% EDTA, washed twice with calcium/magnesium-free PBS, and resuspended at 1×10^6^ cells/ml in ice-cold DMEM supplemented with 2% FBS. Hoechst 33342 (Sigma-Aldrich) was then added at a final concentration of 5 µg/ml, prior to incubation for 90 min at 37°C with mixing. In some experiments, a subset of cells was incubated with 50 µM verapamil for 30 min at 37°C before addition of Hoechst 33342 to examine whether this treatment blocked the efflux of fluorescent Hoechst 33342 from the sorted cells [22]. The cells were then washed twice with PBS, and resuspended in medium containing 2 µg/ml propidium iodide (Sigma-Aldrich), followed by incubation for 30 min at 4°C in the dark. Dual-wavelength analysis was performed to analyze cell sub-populations by FACS.

### Cell invasion assay

Cell invasion was performed using a BD BioCoat Matrigel Invasion Chamber (Becton-Dickinson). Parental and sorted cells were suspended in DMEM containing 1% FBS, before incubation into the upper chamber at a density of 1×10^5^ cells/well. Cell invasion into Matrigel was determined after 24 h of culture at 37°C. The invading cells found in the membrane were fixed using ice-cold methanol, and stained with 0.01% crystal violet. Cell invasion was quantified using the Leica DM IL Inverted Contrasting Microscope after removing non-invading cells on the upper side of the membrane with cotton swabs.

### Tumor formation in mice

NOD/SCID mice were purchased from the animal institute of Tzu-Chi University (Taiwan), and were maintained individually in micro-isolator cages under controlled temperature (24±2°C), humidity (60±10%) and alternating 12-h light/dark cycles. All experiments were carried out in strict accordance with the recommendations of the National Institutes of Health and approved by the Institutional Animal Care and Use Committee (IACUC) of Taiwan (Permit Number: 09-TACTRI-IACUC-014). Freshly-sorted cells suspended in 200 µl PBS containing 10% FBS were inoculated into the axillary fossa of 6- to 7-week-old NOD/SCID mice at a dose of 100, 500, or 1,000 cells per mouse on the same afternoon that the cells were sorted. The mice were monitored twice weekly for palpable tumor formation, and were euthanized 12 weeks after cell inoculation to assess tumor formation. The mice were photographed, and a portion of the subcutaneous tissue at the site of cell injection was collected, fixed in 10% formaldehyde, and embedded in paraffin for H&E staining.

### RNA extraction and quantitative real-time RT-PCR

Total RNA was isolated with Trizol (Invitrogen). First-strand cDNA was reverse-transcribed (RT) according to the manufacturer protocols. Relative levels of mRNA were determined by q-PCR using a real-time PCR system. Several stem cell genes and epithelial-mesenchymal transition markers were analyzed. The primer sequences used are shown in Table 2. β-actin was used as reference. cDNA was subjected to PCR for initial denaturation at 95°C for min, followed by 50 cycles of 95°C for 30 sec, 60°C for 30 sec, and 72°C for 20 sec, and terminal extension at 72°C for 7 min. q-PCR was performed using a Roche LightCycler 480 System (Roche Diagnosis, Mannheim, Germany).

**Table 2:** DNA primers used for q-RT-PCR analysis in this study.

Gene	Forward primer (5′-3′)	Reverse primer (5′-3′)
*Sox-2*	ACACCAATCCCATCCACACT	GCAAACTTCCTGCAAAGCTC
*4-Oct*	GCAATTTGCCAAGCTCCTGAA	GCAGATGGTCGTTGGCTGA
*Nanog*	TTCCTTCCTCCATGGATCTG	TCTGCTGGAGGCTGAGGTAT
*Bmi-1*	ATGCATCGAACAACGAGAATCAAGATCACT	TCAACCAGAAGAAGTTGCTGATGACCC
*Rex-1*	TGGACACGTCTGTGCTCTTC	GTCTTGGCGTCTTCTCGAAC
*ABCG2*	GGGTTCTCTTCTTCCTGACGACC	TGGTTGTGAGATTGACCAACAGAC
*MMP2*	ACGACCGCGACAAGAAGTAT	ATTTGTTGCCCAGGAAAGTG
*MMP9*	CCCTGGAGACCTGAGAACCA	CCCGAGTGTAACCATAGCGG
*E-cadherin*	ATTTTTCCCTCGACACCCGAT	TCCCAGGCGTAGACCAAGA
*β*-*actin*	GGATCTTCATGAGGTAGTCAG	GAGACCTTCAACACCCCAGCC

### Western blotting analysis

Cells were lysed with RIPA lysis buffer (Millipore) in the presence of protease and phosphatase inhibitors (P2580, Sigma-Aldrich), and the concentration of total cellular protein was quantified using the Bradford reagent as instructed by the manufacturer (Bio-Rad, Hercules, CA). In addition, the nuclear and cytosolic protein fractions were prepared with NE-PER Nuclear and Cytoplasmic Extraction Reagents (Thermo Scientific, Waltham, MA). Western blotting analysis was performed with 30 µg proteins of cell lysates separated on 12% SDS-PAGE gel. Proteins transferred to PVDF membrane (Millipore) were probed with monoclonal or polyclonal antibodies specific to p-GSK-3β (Ser-9), GSK-3β, β-catenin, β-actin, MMP-2, ABCG2, lamin-B1 (Santa Cruz Biotechnology, Santa Cruz, CA), or p-β-catenin (Cell Signaling Technology, Danvers, MA; product #9565). Immobilon Western Chemiluminescent HRP Substrate (Millipore) was used for the detection of primary antibody-probed protein bound with an appropriate horseradish peroxidase-conjugated secondary antibody.

### Statistical analysis

SPSS11.0 and Excel 2007 were used for data processing and for analyzing the significance between parental and sorted cells using the unpaired Student *t*-test. Data were expressed as the mean ± SD from at least three independent experiments. *P* values<0.05 were considered statistically significant.

## Supporting Information

Figure S1 **CD24^+^ cells show enhanced sphere formation in HK1 and TW076 cell lines.** Sphere formation in parental, CD24^+^ and CD24^−^ cells in HK1 and TW076 cell lines cultured in DMEM supplemented with 20 ng/ml bFGF and 20 ng/ml EGF for 30 days. The images are representative results of three independent experiments. Scale bars: 100 µm.
(TIF)

Figure S2 **Flow cytometry analysis of CD24^+^ and CD44^+^ sub-population in HK1 and TW076 cell lines.** A total of 1×10^6^ cancer cells were collected and stained with anti-human CD24-fluorescein isothiocyanate (FITC) and/or anti-human CD44-phycoerythrin (PE) antibodies. Isotype-matched human antibodies were used as negative control.
(TIF)

Figure S3 **Expression level of stem cell genes in CD24^+^ HK1 and TW076 cell lines.** The mRNA expression level of *Sox2*, *Oct4*, *Nanog*, *Bmi-1* and *Rex-1* in parental, CD24^+^ and CD24^−^ cells from the NPC cell lines HK1 (top panel) and TW076 (bottom panel) was analyzed by quantitative RT-PCR. The results shown represent the average of three independent experiments. *:*p*<0.05, **: *p*<0.01, ***: *p*<0.001.
(TIF)
