# Correction: Identification of CD24 as a Cancer Stem Cell Marker in Human Nasopharyngeal Carcinoma

**DOI:** 10.1371/journal.pone.0109495

**Published:** 2014-09-26

**Authors:** The PLOS ONE Staff

The first three panels of Figure 7C are incorrectly shown as duplicates of the second three panels. The correct version of Figure 7 can be viewed below.

**Figure 7:**
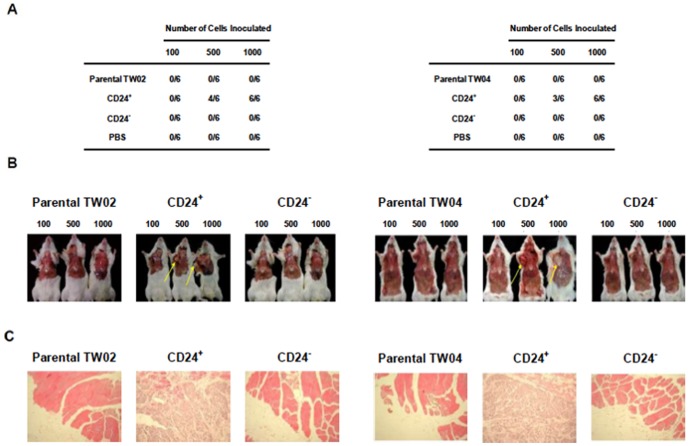
A low number of CD24+ NPC cells initiates tumor formation in NOD/SCID mice. (A) Formation of tumors following injection of CD24+ cells. Groups of six NOD/SCID mice were injected with 100, 500, or 1,000 freshly-sorted CD24^+^ or CD24^−^ cells from the TW02 (left) or TW04 (right) cell line. Mice injected with PBS were used as a negative control. Tumor formation was assessed 12 weeks after cell inoculation. (B) Mice injected with TW02 (left) or TW04 (right) cells were sacrificed for evaluation of tumor formation twelve weeks after inoculation. The arrows indicate the presence of tumors in mice injected with 500 or 1,000 CD24^+^ cells. (C) Tissue H&E staining results of TW02 (left) and TW04 (right) mice inoculated with 500 cells. Inoculation of as few as 500 CD24^+^cells produced histological signs of tumors at the site of injection.
