# Expression of Concern: Identification of CD24 as a Cancer Stem Cell Marker in Human Nasopharyngeal Carcinoma

**DOI:** 10.1371/journal.pone.0210304

**Published:** 2019-01-03

**Authors:** The PLOS ONE Editors

After publication of this article [1], the authors contacted the journal to correct image duplications in Fig 7C; the duplications were not present in the original submission to the journal, but they were present in the revised figure prepared during resubmission. These duplications were addressed in a previous Correction [2].

Soon before the Correction of Fig 7C was published, questions were raised regarding image duplications in Fig 7B. Specifically, similarities were noted between the images shown for the following experimental conditions:

- Parental TW02, 500 and TW02/CD24-, 500
- Parental TW02, 1000 and TW02/CD24-, 1000
- Parental TW04, 100 and TW04/CD24-, 100
- TW04/CD24+, 100 and TW04/CD24-, 500

The authors provided an updated version of this figure in which the TW02 CD24-, TW04 parental, and TW04 CD24- panels are replaced with different images. The authors also provided images of mice from the original experiment (see S1 File), but some images are no longer available. Chang Gung University looked into this matter and clarified that, while the authors retained the lab notebooks as well as some raw materials and data for this experiment (i.e., paraffin-embedded tissues and images of mice), the information found in the lab notebooks was incomplete and some images of mice for Fig 7B are no longer available. Hence, we have been unable to fully clarify the issues about the published figure [1].

When the authors contacted the journal to correct Fig 7B, they also notified the journal of an additional duplication affecting Fig 2B and 8B. Namely, the same blot was included in Fig 2B as the TW02 Lamin B1 control blot (nuclear fraction), and in Figure 8B as the TW04 β-actin blot. The authors explained that there was an error in preparing Fig 2. The original blots underlying the Fig 2B experiments are no longer available, but the authors did an independent replication experiment for the TW02 nuclear fractions. In the revised Fig 2B included with this notice, Lamin B1 and β-catenin blots from the replication experiments replace the previous panels, and the quantification data in the bar graph below are updated accordingly. S2 File includes the raw blots from the replication experiments, and S3 File includes quantification data underlying the corrected bar graph.

Please see the corrected Figs 2 and 7, and corrected Fig 2 caption, here.

**Fig 2:**
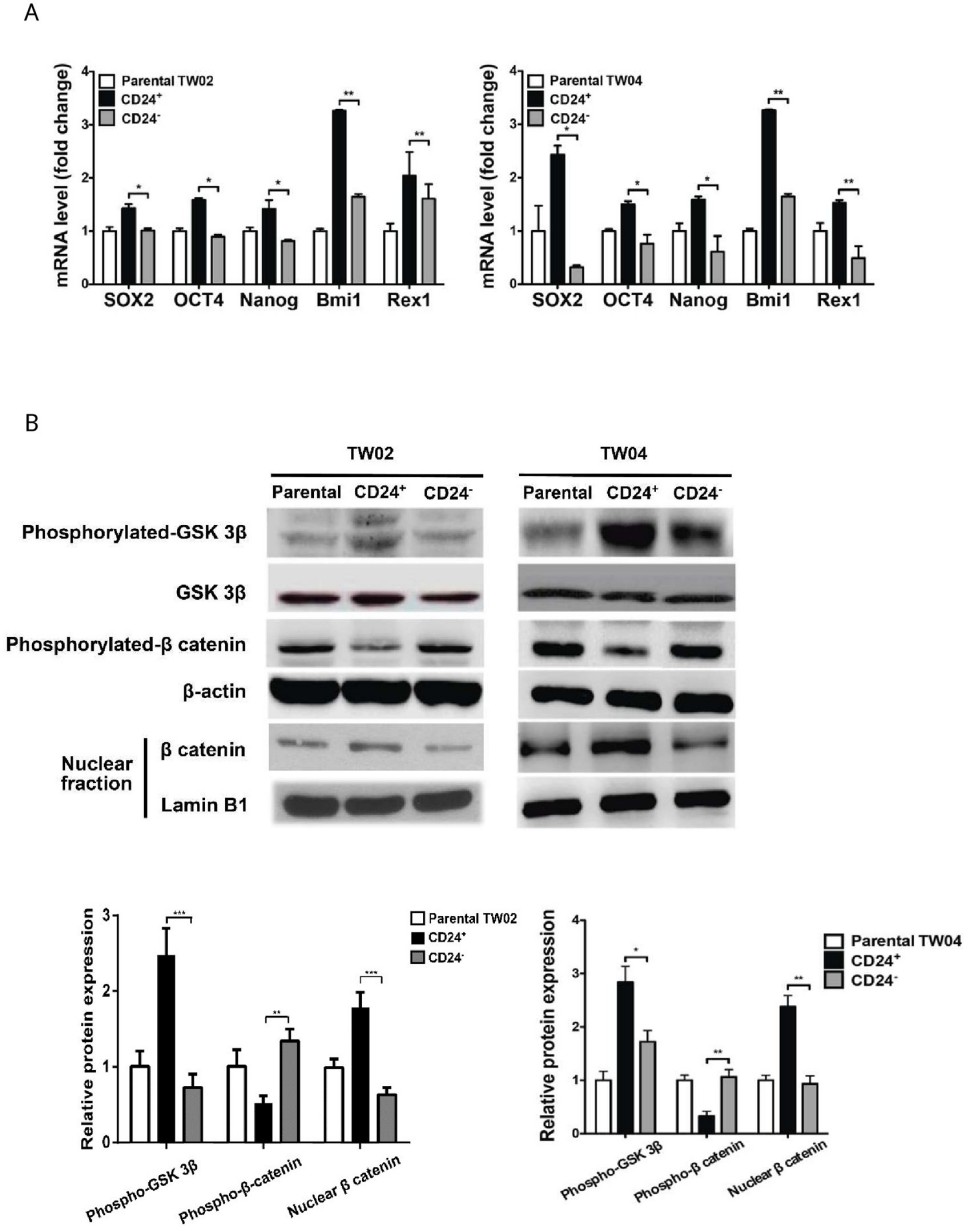
Expression levels of stem cell genes and activation of the Wnt/β-catenin pathway in CD24+ cells. (A) mRNA expression of *Sox2*, *Oct4*, *Nanog*, *Bmi-1* and *Rex-1* in parental, CD24+ and CD24− cells from the NPC cell lines, TW02 (left) and TW04 (right), was evaluated using quantitative RT-PCR analysis. The results shown represented averages of three independent experiments. *: *p*<0.05, **: *p*<0.01. (B) Western blot analysis of phosphorylated-GSK 3β, GSK 3β, phosphorylated-β-catenin and β-actin in whole cell lysates, and of β-catenin and lamin B1 in nuclear fractions of parental, CD24+, and CD24− cells isolated from the TW02 and TW04 cell lines. Quantitative results were calculated using the ImageJ software. The TW02 nuclear fractions correspond to the results of a replication experiment performed after the other experiments reported in the original figure. New samples were generated for this replication experiment, but the same methodology reported in the original article was used. The results represent means ± SD of three independent trials. Protein expression levels for phosphorylated-GSK 3β, phosphorylated-β catenin and nuclear β catenin were normalized against non-phosphorylated GSK 3β, β-actin and nuclear lamin B1, respectively. *: *p*<0.05, **: *p*<0.01, ***: *p*<0.001.

**Fig 7:**
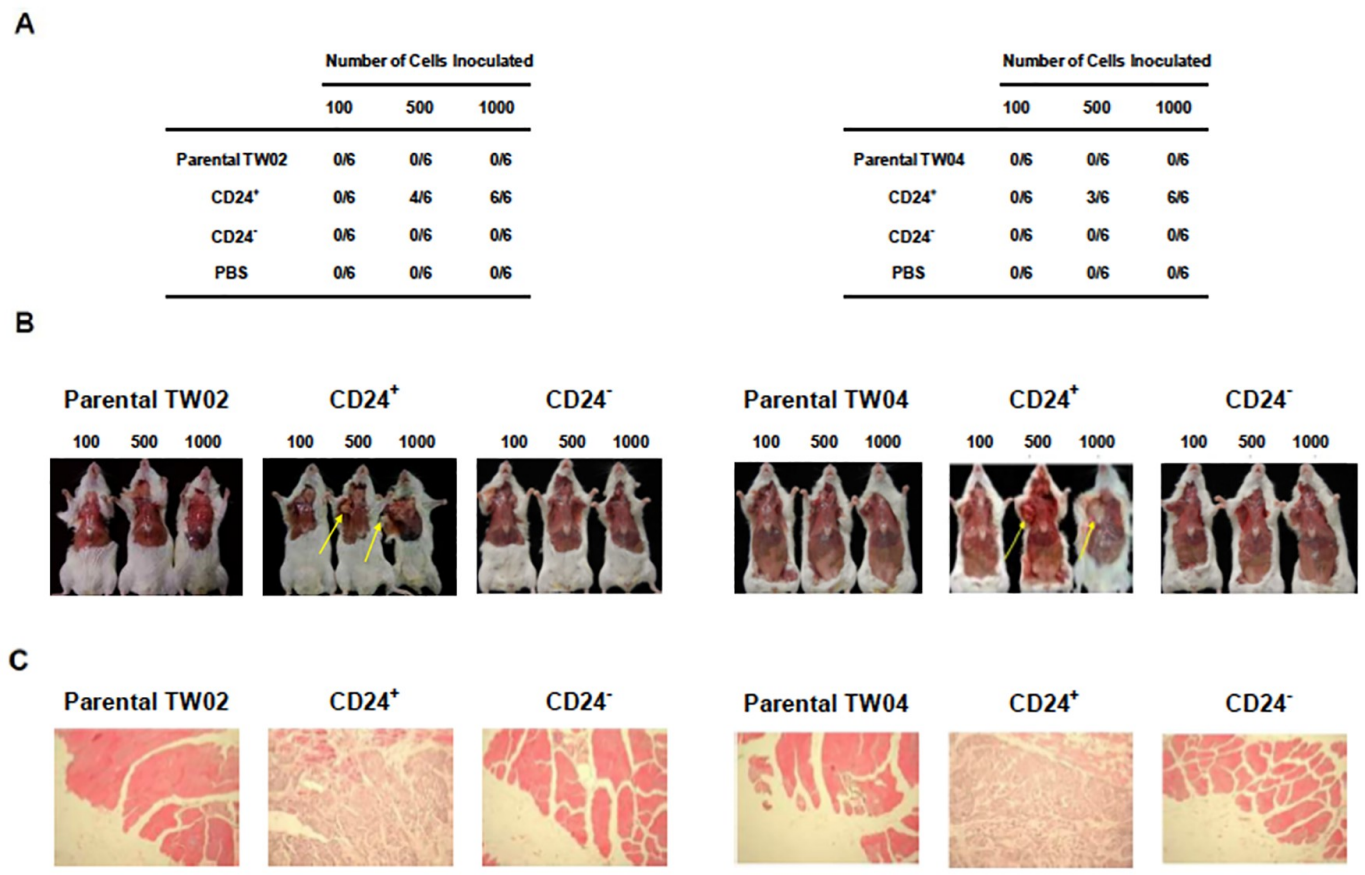
A low number of CD24+ NPC cells initiates tumor formation in NOD/SCID mice. (A) Formation of tumors following injection of CD24+ cells. Groups of six NOD/SCID mice were injected with 100, 500, or 1,000 freshly-sorted CD24+ or CD24− cells from the TW02 (left) or TW04 (right) cell line. Mice injected with PBS were used as a negative control. Tumor formation was assessed 12 weeks after cell inoculation. (B) Mice injected with TW02 (left) or TW04 (right) cells were sacrificed for evaluation of tumor formation twelve weeks after inoculation. The arrows indicate the presence of tumors in mice injected with 500 or 1,000 CD24+ cells. (C) Tissue H&E staining results of TW02 (left) and TW04 (right) mice inoculated with 500 cells. Inoculation of as few as 500 CD24+ cells produced histological signs of tumors at the site of injection.

We regret that the journal did not correct Figs 2B and 7B sooner. While the authors stand by the validity of the experimental data and the conclusions reached in the study, they were unable to provide all of the original images for the mouse experiments, as described above. In light of the image duplications and the unavailability of primary data, the *PLOS ONE* Editors have concerns about the strength of evidence supporting the in vivo claims made in the article and, therefore, issue this Expression of Concern.

## Supporting information

S1 File Raw data of Fig 7B. (PDF)

S2 File Raw data for the repeated Western blot of corrected Fig 2B. (PDF)

S3 File Protein expression levels for corrected Fig 2B. (XLSX)
