# Supplementary figures and images for: Identification of CD24 as a Cancer Stem Cell Marker in Human Nasopharyngeal Carcinoma
Source: PLoS One. 2014 Jun 23;9(6):e99412. doi: 10.1371/journal.pone.0099412 (PMC4067285; doi:10.1371/journal.pone.0099412)

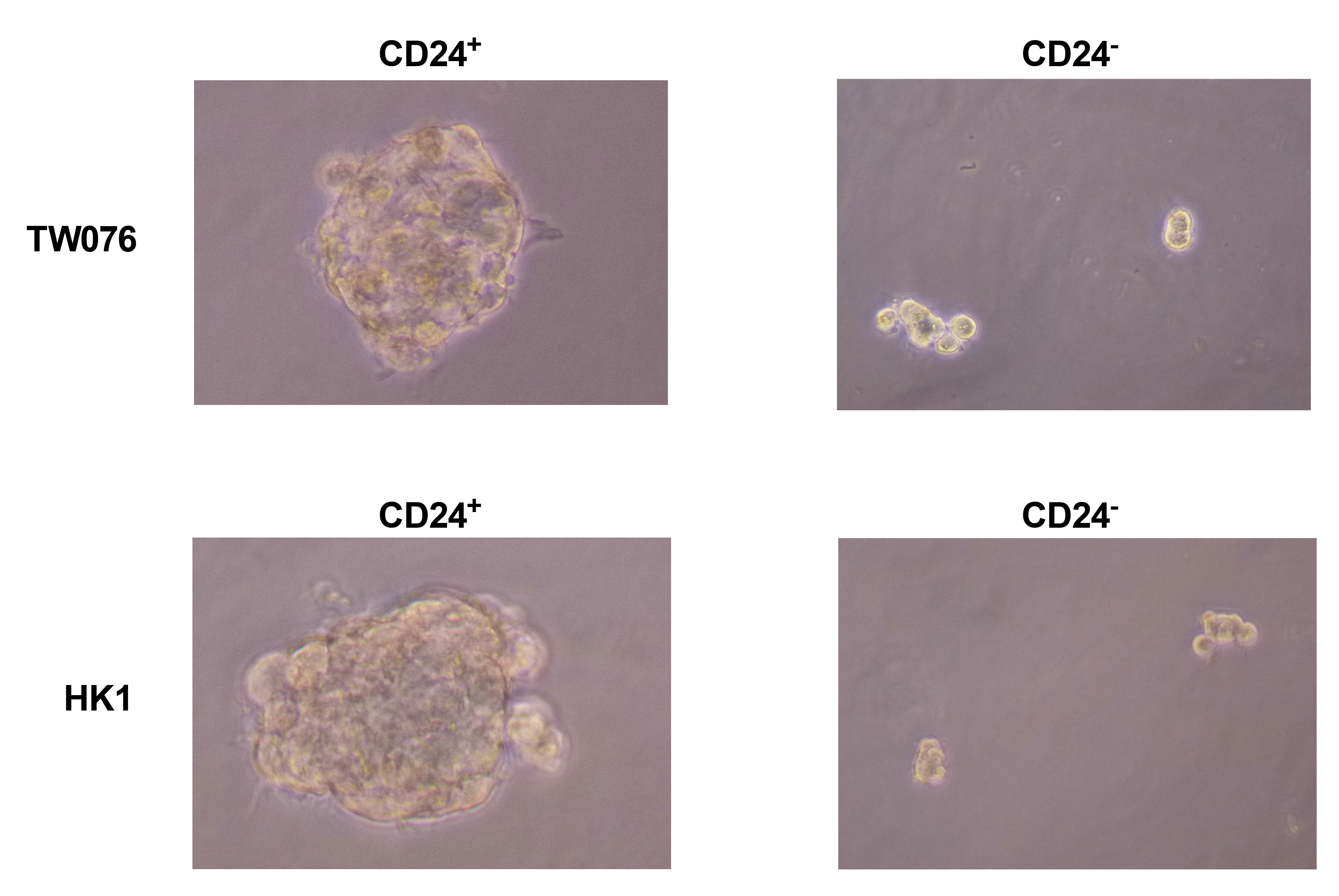

Supplement: Figure S1 — CD24+ cells show enhanced sphere formation in HK1 and TW076 cell lines. Sphere formation in parental, CD24+ and CD24− cells in HK1 and TW076 cell lines cultured in DMEM supplemented with 20 ng/ml bFGF and 20 ng/ml EGF for 30 days. The images are representative results of three independent experiments. Scale bars: 100 µm. (TIF) [file pone.0099412.s001.tif]

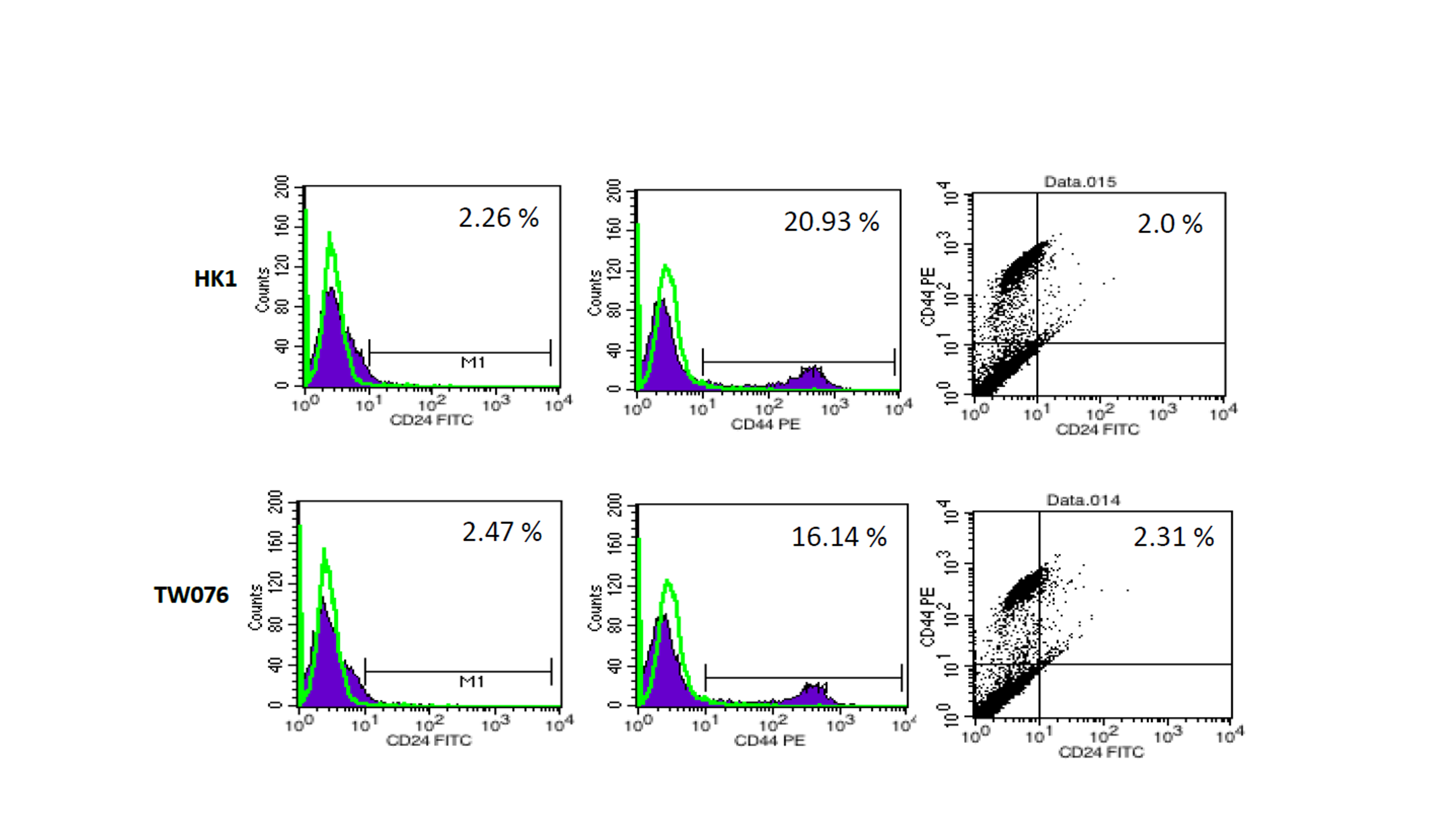

Supplement: Figure S2 — Flow cytometry analysis of CD24+ and CD44+ sub-population in HK1 and TW076 cell lines. A total of 1×10^6^ cancer cells were collected and stained with anti-human CD24-fluorescein isothiocyanate (FITC) and/or anti-human CD44-phycoerythrin (PE) antibodies. Isotype-matched human antibodies were used as negative control. (TIF) [file pone.0099412.s002.tif]

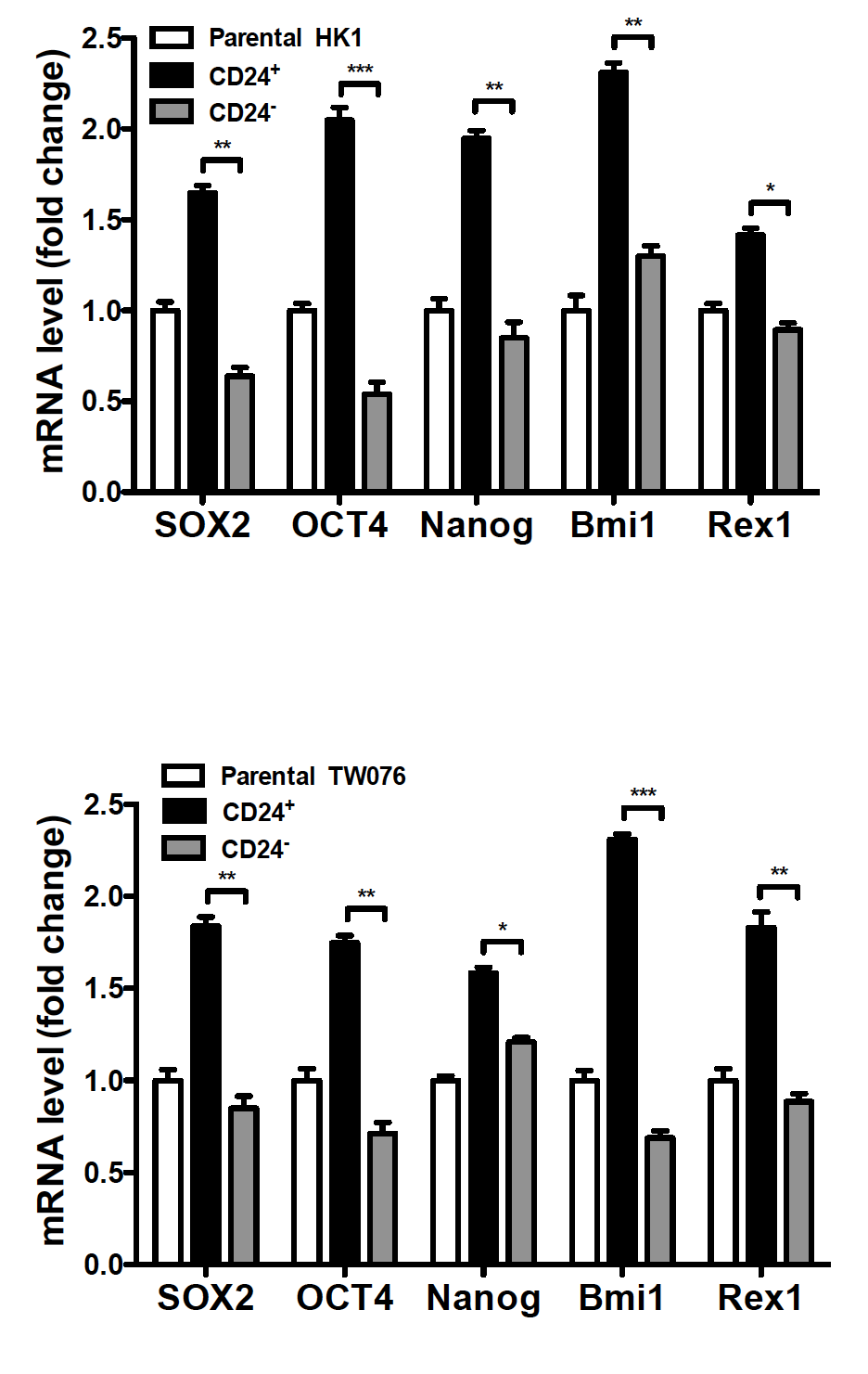

Supplement: Figure S3 — Expression level of stem cell genes in CD24+ HK1 and TW076 cell lines. The mRNA expression level of Sox2, Oct4, Nanog, Bmi-1 and Rex-1 in parental, CD24+ and CD24− cells from the NPC cell lines HK1 (top panel) and TW076 (bottom panel) was analyzed by quantitative RT-PCR. The results shown represent the average of three independent experiments. *:p<0.05, **: p<0.01, ***: p<0.001. (TIF) [file pone.0099412.s003.tif]
