# Supplementary material for: Expression of Concern: Identification of CD24 as a Cancer Stem Cell Marker in Human Nasopharyngeal Carcinoma
Source: PLoS One. 2019 Jan 3;14(1):e0210304. doi: 10.1371/journal.pone.0210304 (PMC6317794; doi:10.1371/journal.pone.0210304)
Supplement: S1 File — (PDF) [file pone.0210304.s001.pdf]

File S1. Raw data of Figure 7B

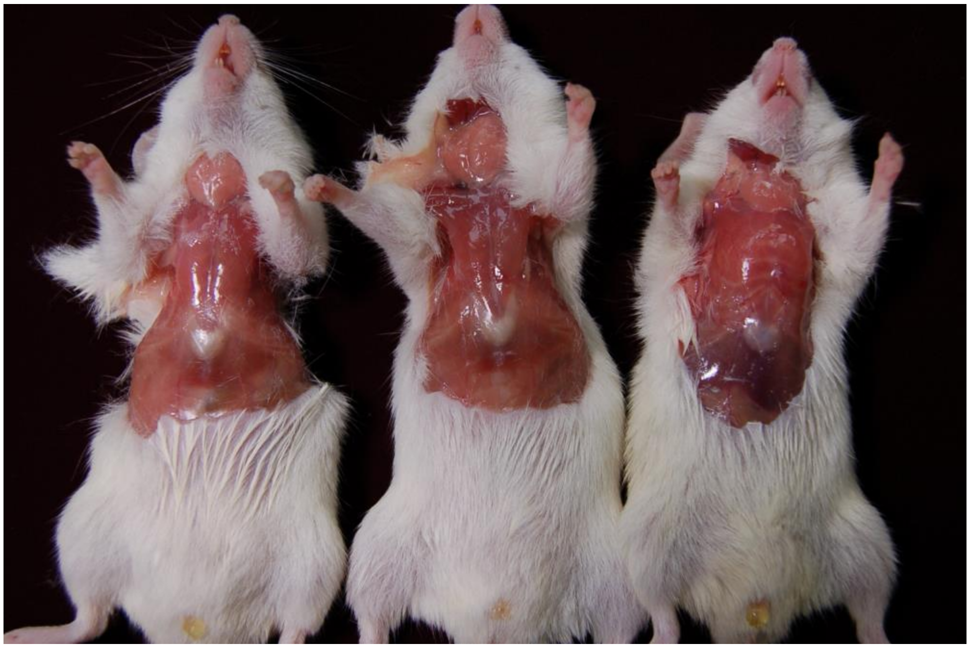

01. TW02 Parental (100, 500, 1000 cells)

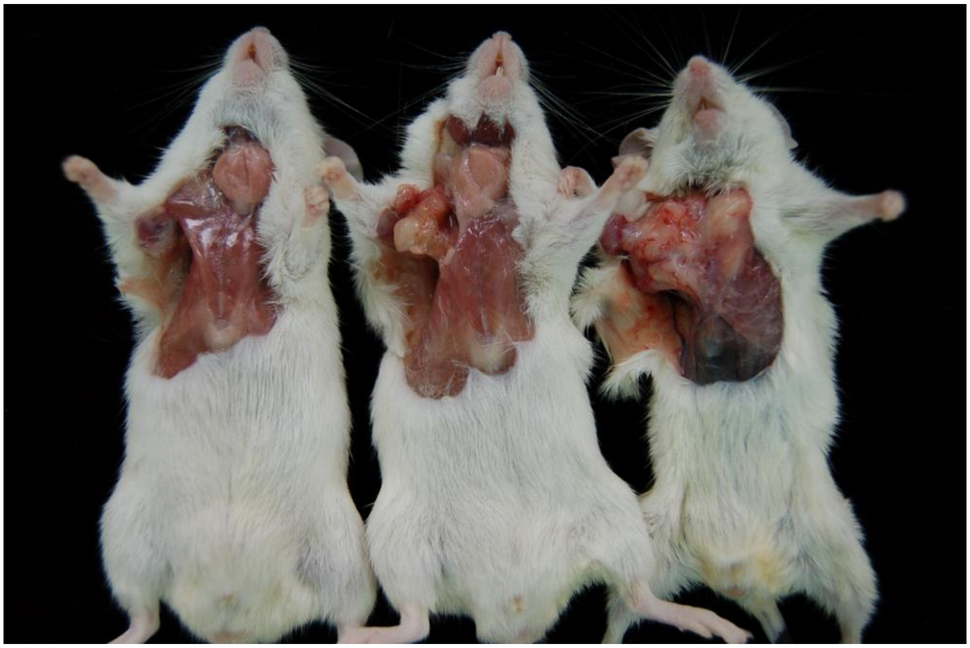

02. TW02 CD24+ (100, 500, 1000 cells)

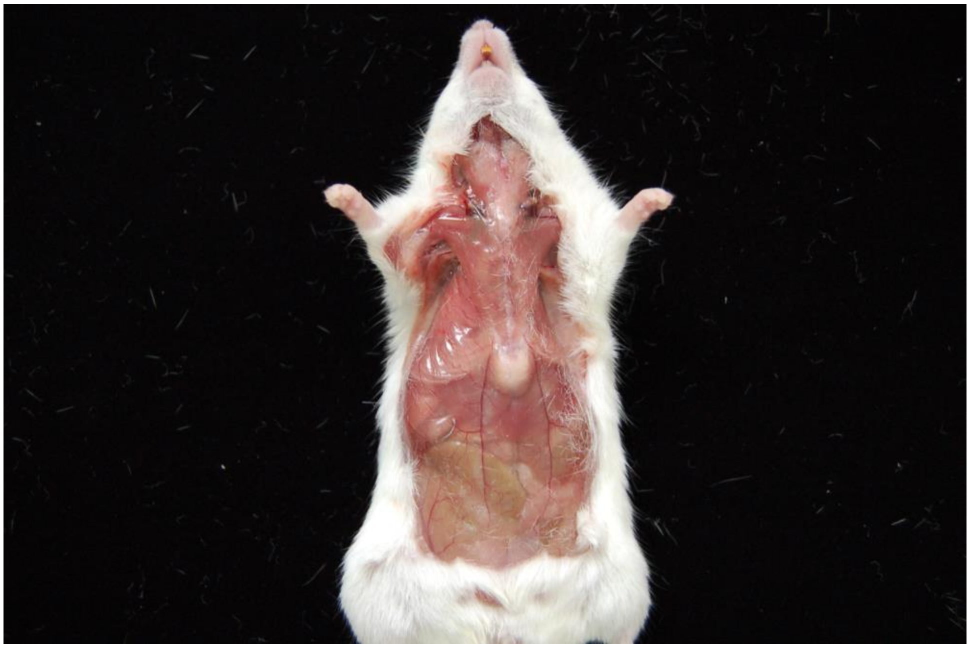

03. TW04 CD24+ (100 cells)

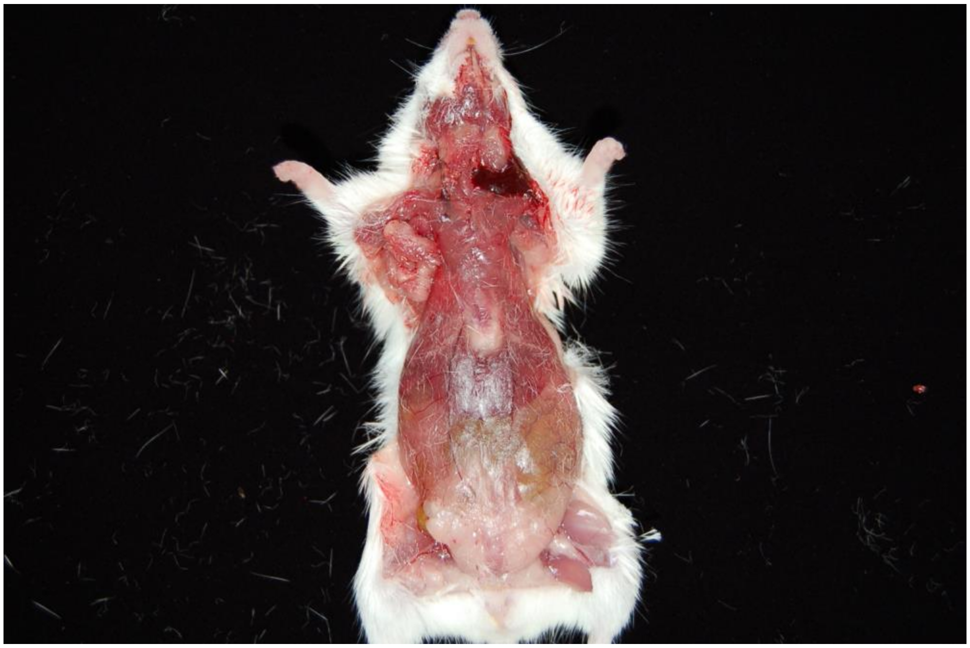

04. TW04 CD24+ (500 cells)

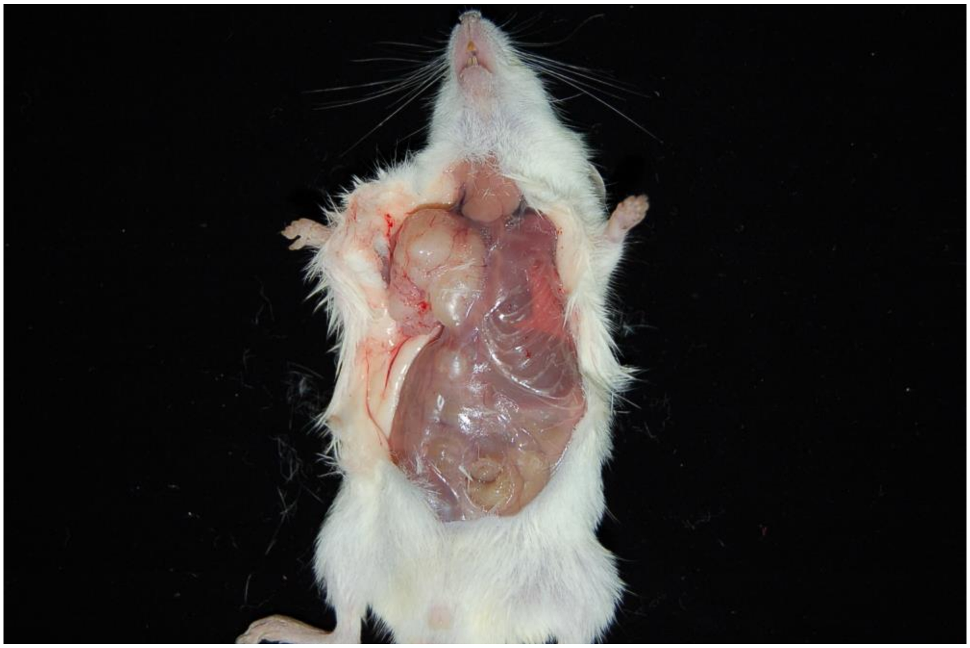

05. TW04 CD24+ (1000 cells)

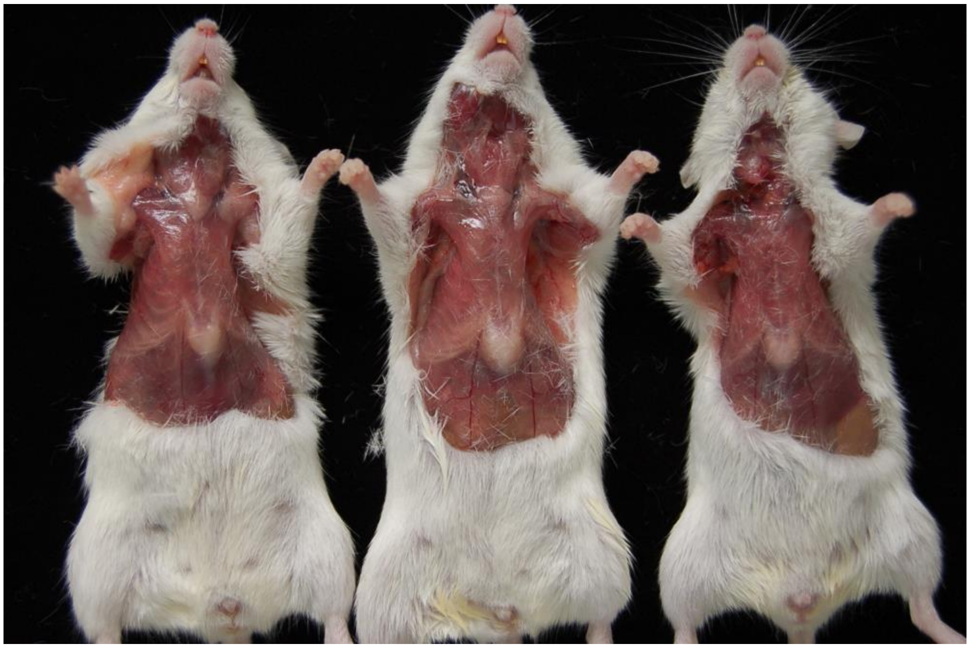

06. TW02 CD24- (100, 500, 1000 cells)

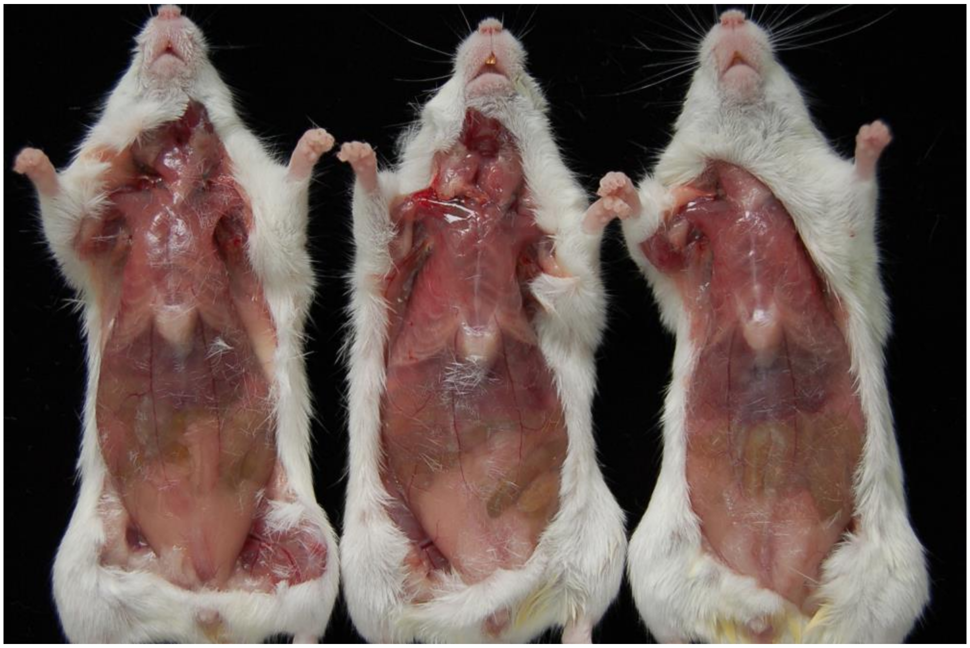

07. TW04 Parental (100, 500, 1000 cells)

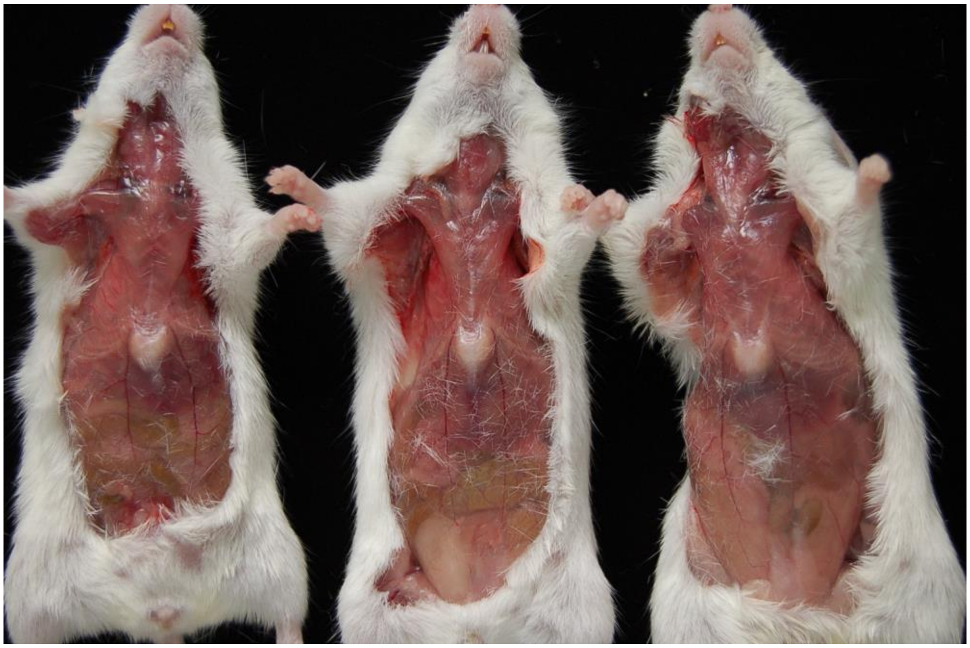

08. TW04 CD24- (100, 500, 1000 cells)

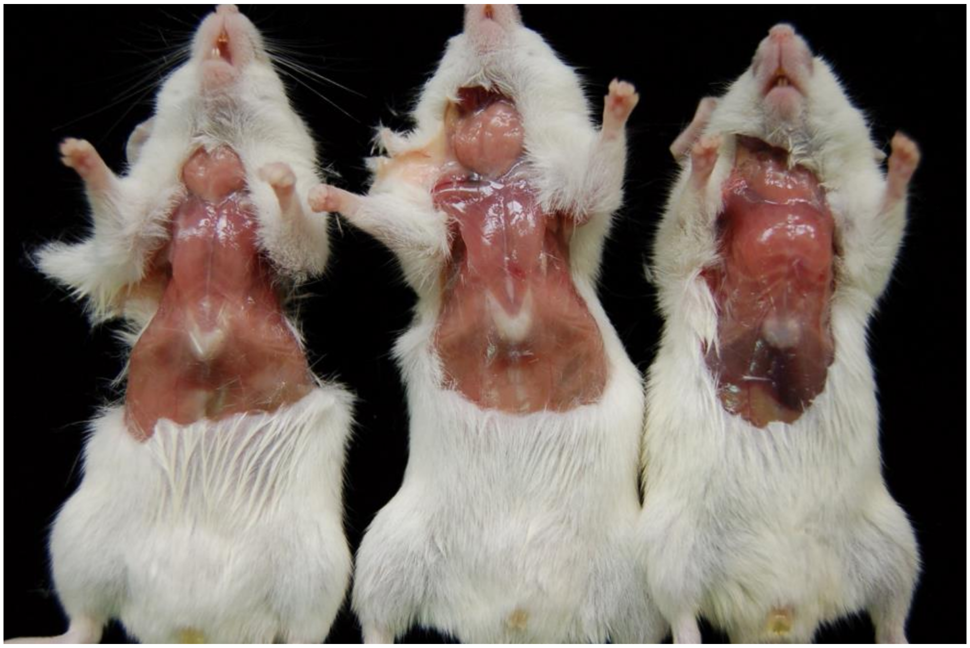
09. Parental TW02 (100, 500, 1000 cells)

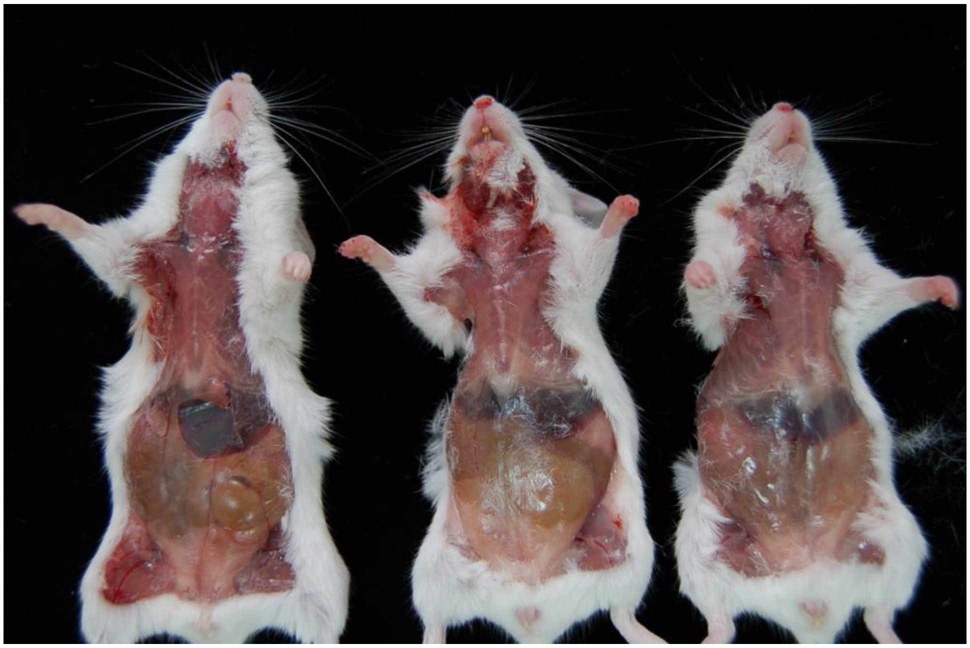
10. Parental TW04 (100, 500, 1000 cells)

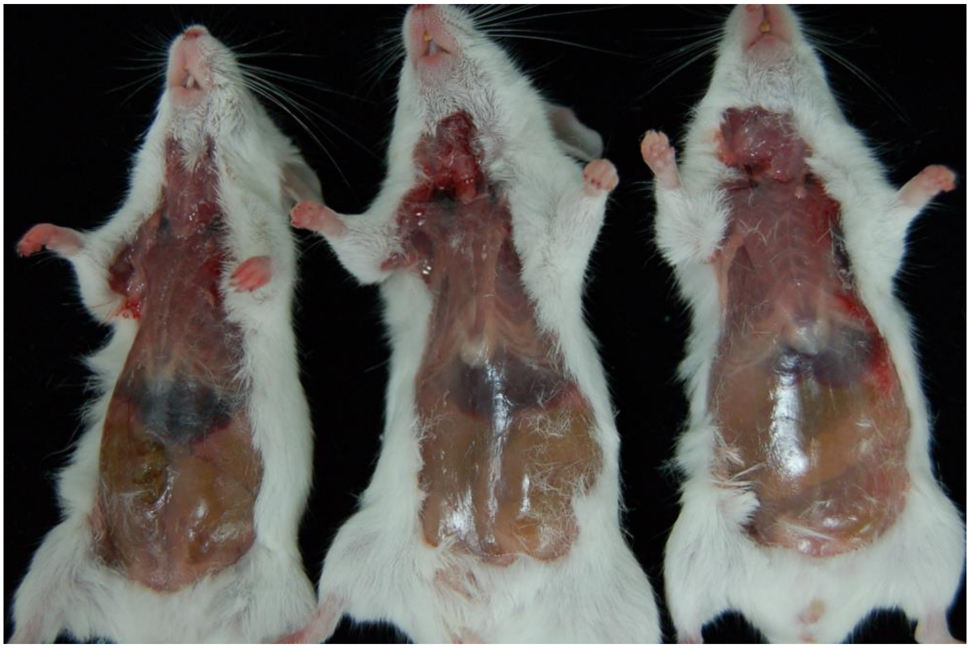

11. TW04 CD24- (100, 500, 1000 cells)
