# Supplementary material for: Expression of Concern: Identification of CD24 as a Cancer Stem Cell Marker in Human Nasopharyngeal Carcinoma
Source: PLoS One. 2019 Jan 3;14(1):e0210304. doi: 10.1371/journal.pone.0210304 (PMC6317794; doi:10.1371/journal.pone.0210304)

Correction for article e99412

Raw data for repeated Western blot experiment of β-catenin and lamin B1 in panel TW02 (Figure 2B):

β-catenin

Parental CD24$^+$ CD24$^-$

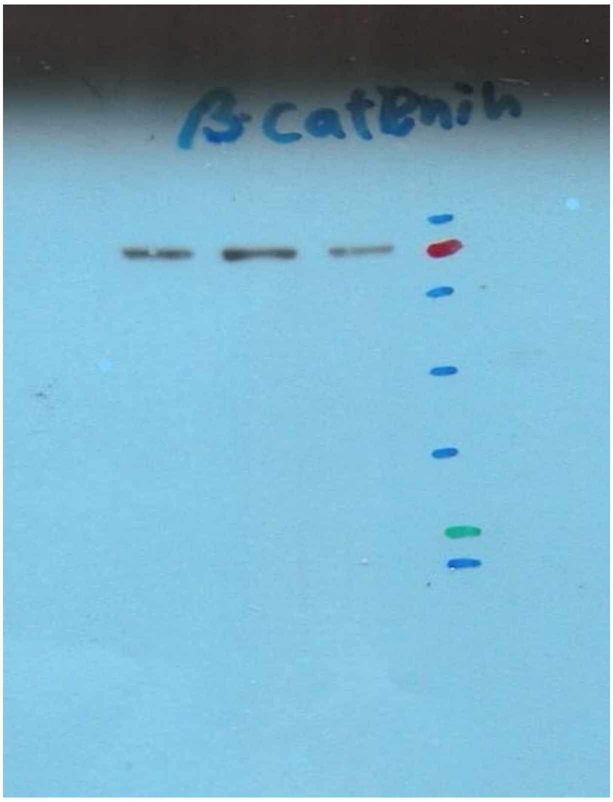

Lamin B1

Parental CD24$^+$ CD24$^-$

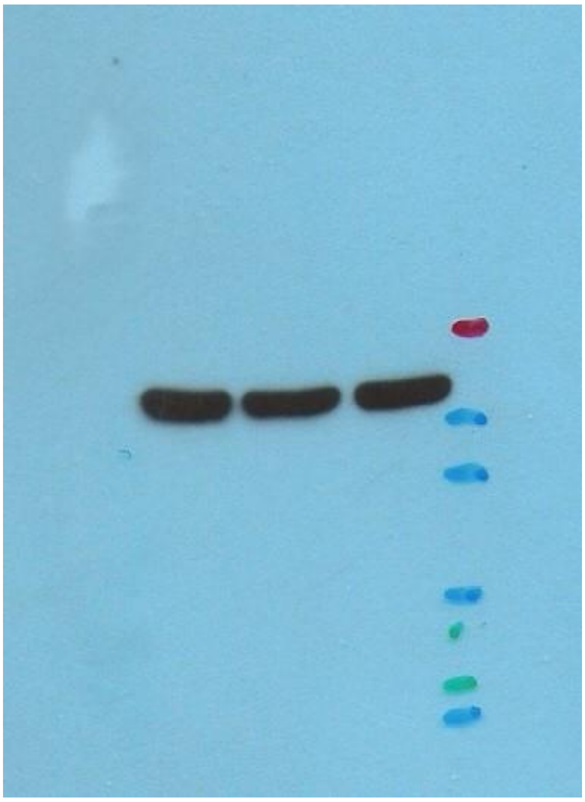

Supplement: S2 File — (PDF) [file pone.0210304.s002.pdf]
